# State-of-the-art in artificial neural network applications: A survey

**DOI:** 10.1016/j.heliyon.2018.e00938

**Published:** 2018-11-23

**Authors:** Oludare Isaac Abiodun, Aman Jantan, Abiodun Esther Omolara, Kemi Victoria Dada, Nachaat AbdElatif Mohamed, Humaira Arshad

**Affiliations:** a School of Computer Sciences, Universiti Sains Malaysia, Penang, Malaysia; b Department of Computer Science, Bingham University, Karu, Nigeria; c Department of Computer Science, Ahmadu Bello University, Zaria, Nigeria; d Department of Mathematical Sciences, Nasarawa State University, Keffi, Nigeria; e California University, USA; f Department of Computer Science and Information Technology, Islamia University of Bahawalpur, Pakistan

**Keywords:** Computer science

## Abstract

This is a survey of neural network applications in the real-world scenario. It provides a taxonomy of artificial neural networks (ANNs) and furnish the reader with knowledge of current and emerging trends in ANN applications research and area of focus for researchers. Additionally, the study presents ANN application challenges, contributions, compare performances and critiques methods. The study covers many applications of ANN techniques in various disciplines which include computing, science, engineering, medicine, environmental, agriculture, mining, technology, climate, business, arts, and nanotechnology, etc. The study assesses ANN contributions, compare performances and critiques methods. The study found that neural-network models such as feedforward and feedback propagation artificial neural networks are performing better in its application to human problems. Therefore, we proposed feedforward and feedback propagation ANN models for research focus based on data analysis factors like accuracy, processing speed, latency, fault tolerance, volume, scalability, convergence, and performance. Moreover, we recommend that instead of applying a single method, future research can focus on combining ANN models into one network-wide application.

## 1 Introduction

In recent times artificial neural networks (ANNs) has become popular and helpful model for classification, clustering, pattern recognition and prediction in many disciplines. ANNs are one type of model for machine learning (ML) and has become relatively competitive to conventional regression and statistical models regarding usefulness [1]. Currently, artificial intelligence (machine learning, neural network, deep learning, robotic), information security, big data, cloud computing, internet, and forensic science are all hotspots and exciting topics of information and communication technology (ICT). ANNs full applications can be evaluated with respect to data analysis factors such as accuracy, processing speed, latency, performance, fault tolerance, volume, scalability and convergence [2, 3]. The great potential of ANNs is the high-speed processing provided in a massive parallel implementation and this has heightened the need for research in this domain [4]. ANNs can be developed and used for image recognition, natural language processing and so on. Nowadays, ANNs are mostly used for universal function approximation in numerical paradigms because of their excellent properties of self-learning, adaptivity, fault tolerance, nonlinearity, and advancement in input to an output mapping [5].

These data analysis factors give more reason why ANNs are effective, efficient and successful in providing a high level of capability in handling complex and non-complex problems in many spheres of life. ANNs are capable of handling problems in agriculture, science, medical science, education, finance, management, security, engineering, trading commodity and art. Including problems in manufacturing, transportation, computer security, banking, insurance, properties management, marketing, energy, and those challenges that cannot be solve by the computational ability of traditional procedures and conventional mathematics. Despite these extensive applications of ANNs, there is an increasing need to address the problem of adopting a systematic approach in ANNs development phase to improve its performance. For instance, an approach to address major factors and topics in a choice of data sets (size, volume, small, large and otherwise), the accuracy of data, data instrument, data standardization, type of data inputs, data division, and data preprocessing, validations, processing and output techniques.

Also, other key challenges or issues that are common with ANN modeling which have received interest and require further investigation in future. Including developmental techniques that can improve designing of robust models, improving pattern transparency and allowing useful knowledge from trained ANNs. More also is the challenges of improving extrapolation ability, new approaches to uncertainty and improving convergences. More also, there is continuous gradient enigma and quantization of variable problems and noise. Furthermore, there is a need to address the traversal of the error surface by utilizing quantization of variable and time-consuming convergence problems common to most artificial neural systems (ANS) that use supervised training. Some of these problems are highlights as follows:

(i) Improve designing of robust models: model robustness means predictive capability of ANN kinds in generalizing range of data like those used for training. An example is using of textual data or information to improve modeling prediction of the financial market. Some experts believe if that ANNs become globally accepted and reach apex potentiality, they will not only provide a good fit to calibration and validation of data. But will enable predictions that will be plausible regarding model's correlation and robustness in any range of conditions [6, 7]. ANNs validated of error can give accurate predictions for conditions like those found in trained data.
(ii) Improving of model transparency and the enabling of knowledge extraction from trained ANNs: means the possibility of interpreting ANN models in a way that provides a deep understanding of the effect of model inputs to outputs.
(iii) Improving extrapolation ability: extrapolation of ANN models is the capability of the model to predict accurately outward range of data used for ANN model calibration. ANNs perform best if they do not extrapolate above the range of data used for design or model calibration [8, 9, 10].
(iv) New approaches to uncertainty: another limitation of ANNs including uncertainty in predictions which may not be taken to account. When uncertainty is not accounted, it becomes difficult to measure ANN predictions quality, which can critically limits or reduces their efficacy. Although ANNs has had their issues, new approaches like cognitive computing and deep learning have significantly raised the support in these fields. A synthetic machine might still be out of reach, but systems like ANNs that help improve people's lives are here today.

## 2 Main text

### 2.1 Artificial neural networks

Nowadays ANNs application have become popular in various area of human needs. Many organizations are investing in the neural networks to solve problems in various fields and the economic sector which traditionally fall under the responsibility of operations research [11]. What makes artificial intelligence unique is that it is mostly proposed for data analyses by academics in the fields of social science and arts apart from its usefulness in science and engineering [12], because of its wide applications. For example, in recent times, artificial intelligence (AI) has been extensively applied to optimization issues in diverse areas like industrial production and petroleum exploration [13] and business [14] setting.

**Fig. 1:**
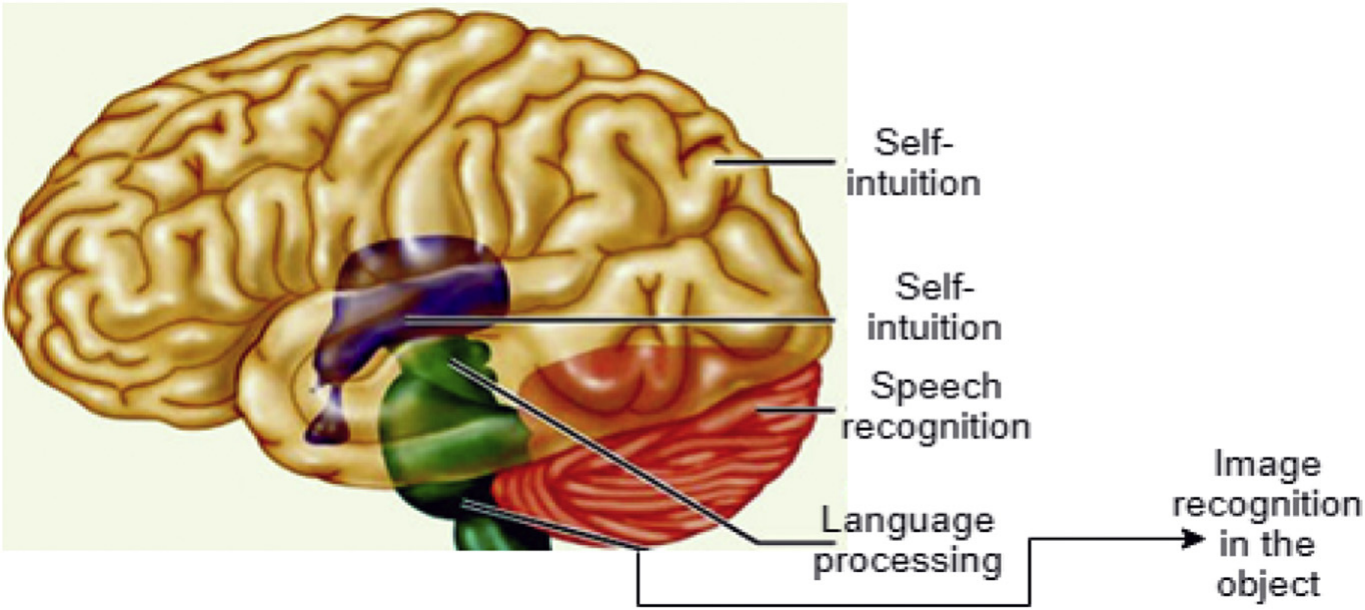
A typical human brain structure with operational capabilities.

A good advantage of ANNs application is that it can make models easy to use and more accurate from complex natural systems with large inputs [15]. The ANN is found to be a very novel and useful model applied to problem-solving and machine learning. ANN is an information manager model that is similar to biological nervous systems function of the man brain. Recently, research interest in brain functionality has rapidly increased globally [16]. According to Haykin [17], an ANN can be comparable machine produced to function the same way the human brain performs a given task of interest. For example, "the human brain is big and highly efficient. The man brain is like an information-processing machine that has a variety of complex signal computing operations” [18], that can be easily coordinated to perform a task. The main element of this brain is the unique design of their information-processing capability. It constitutes many complexes interconnected “neurons” in the form of elements working together to solve specific problems on daily basis. A typical example of a neural network function is the human brain that is connected to send and receive signals for human action. An illustration of how the human brain function is explained in Fig. 1.

**Fig. 2:**
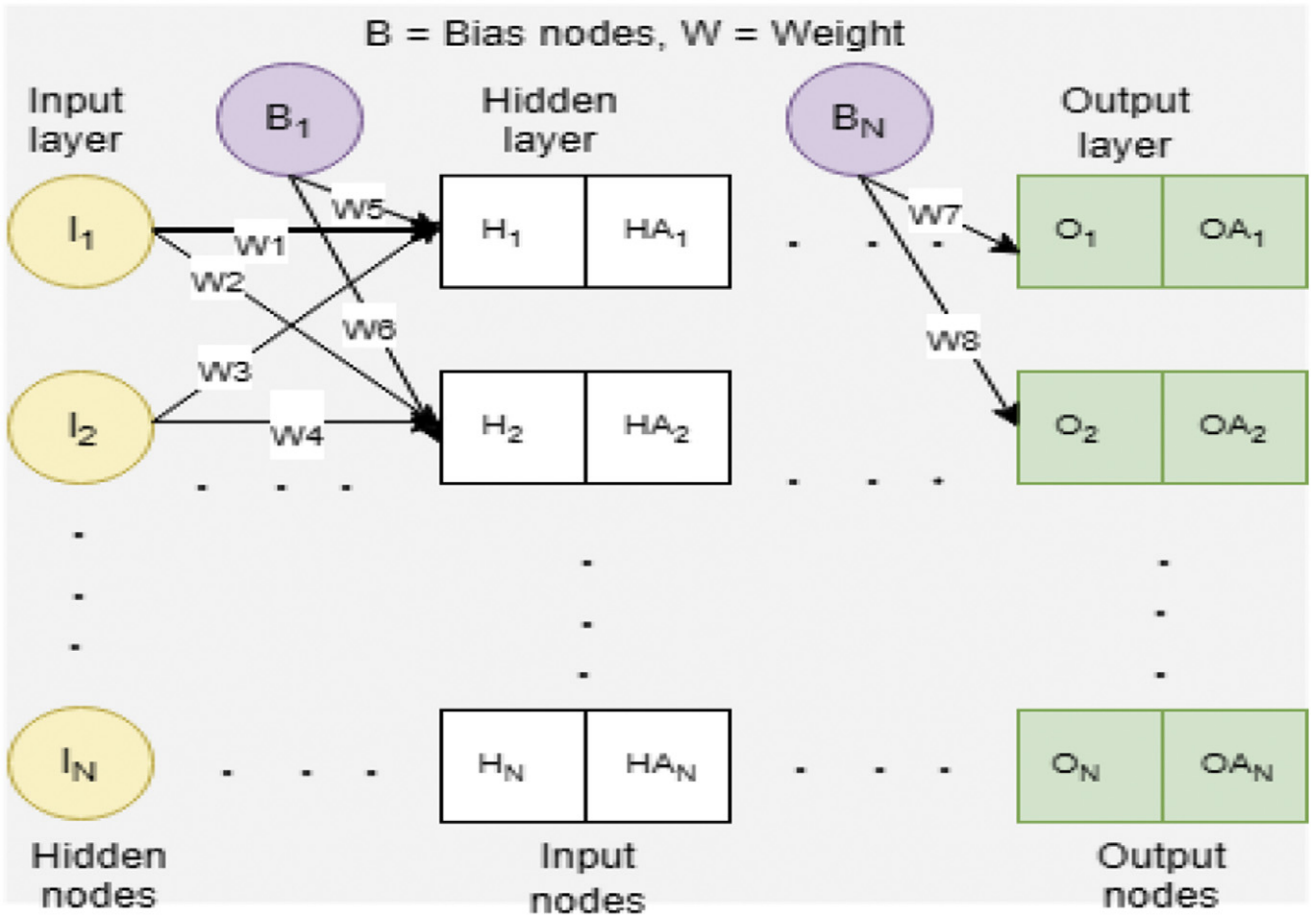
A typical neural network architecture.

Fig. 1 is a demonstration of a connection within the brain working like a neural network that performs intelligence reasoning functions. Brainstorming to understand a scenario (like an internet web search platform), recognizing speech (e.g. from a known person and unknown person) like the human brain, recognizing an image (from an object) like the brain, can process language (translate language) like the human brain does and can perform other things like eating, riding a bicycle (self-intuition). For more specific examples; ANNs has seen massive use in specific domains, such as; diagnosis of hepatitis; speech recognition; recovery of data in telecommunications from faulty software; interpretation of multi-language messages; three-dimensional object recognition; texture analysis; facial recognition; undersea mine detection; and hand-written word recognition. Thus, ANNs can learn by example like people. In some cases, ANNs can be designed for a specific application like data classification or pattern recognition through the learning process [19]. Learning in the human brain requires adjustments to the synaptic relationship between and among neurons, likewise the learning in ANNs [19]. Generally, an ANN function like an imitation of the man brain [1, 20]. An architecture of a typical NN is showed in Fig. 2.

Neural network (NN) layers are independent of one another; that is, a specific layer can have an arbitrary number of nodes. This arbitrary number of nodes is called bias node. The bias nodes always set as equal to one. In analogy, the bias nodes are like the offset in linear regression given as; y = ax + b, where “a” is the coefficient of independent “x” and then “b” is called slope. A bias major function is to provide node with a constant value that is trainable, in addition to the normal inputs received by the network node. Importantly, a bias value enables one to move the activation function either to the right or the left, that can be analytical for ANN training success. When the NN used as a classifier, the input and the output nodes will match input features and output classes. However, when the NN is used as a function approximation, it generally has an input and an output node. However, the number of designed hidden nodes essential greater than those of input nodes.

### 2.2 Applications of neural networks

Given this description of neural networks (NNs), how its work, and their real-world applications and uses, indeed, NNs have wide applied to real-world problem in business, education, economics and in many aspects of life problems. NNs are also applicable to optimization method [21] intrusion detection [22, 23] and data classification [24, 25, 26]. Classification regarded as a form of difficult optimization challenge. Most researchers applied machine learning (ML) techniques in solving classification problem [27, 28]. NNs are excellent identifier of trends in data and patterns [29], they are suited for forecasting and prediction needs including those items listing (with references to Supplementary Table 1).

The list in Supplementary Table 1 based on the successful application of ANN to real-world problems. It summarizes neural networks application in practice which integrated into many areas like modeling, classification, pattern recognition, and prediction. Prediction of financial stability is useful in economic, management and development of any nation, which is beneficiary for analyzing the monetary value of any economy. Moreover, ANNs have been used successfully in the prediction of banks success or failure and stock market estimation. Likewise, it is used extensively in forecasting of weather and climatic change which is helpful in human safety and security of properties such as buildings, environment, installation, houses, and transportation. Furthermore, ANNs applied successfully to different areas of agriculture like remote sensing, particularly in the crop type classification and crop production estimation. Therefore, Supplementary Table 1 provides a general review of the wide scope of problems that this neuro-Intelligence system can currently address.

### 2.3 ANN classification

**Fig. 3:**
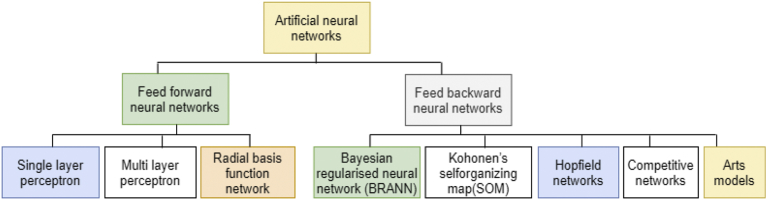
Review framework for artificial neural networks classification.

ANN can be classified as depicted in Fig. 3.

**Fig. 4:**
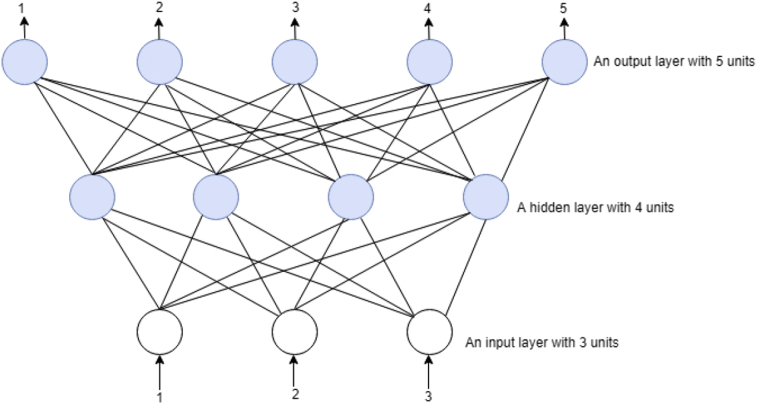
Two-layered feedforward neural network.

A feedforward neural network (FFNN) is a machine learning classification algorithm that made up of organized in layers that are similar to human neuron processing units. In FFNN each unit in a layer relates to all the other units in the layers. These layers connections with units are not all equal because each connection can have a different weight or strength. The weights of the network connections measure the potential amount of the knowledge of the network. Also, NN units are known as *nodes*. The information processing in the network involves data entry from the input units and passes through the network, flowing from one layer to another layer until it gets to the output units. When NN operate normally, that is when its acted as a classifier, then there will be no feedback between layers [30]. In FFNN, information transmitted only in one direction, that is from the input nodes, to the hidden nodes, if any, and then to output nodes. With this behaviour, they are called *feedforward* neural networks [31]. Examples of FFNNs is single layer perception and multilayer perceptron. Example of a two-layered network is 3 input units, 4 units with a hidden layer and 5 units of output layer as circles respectively in Fig. 4.

Fig. 4, has 3 input units as shown in circles, but input units is not part of any layer in the network system, sometimes the input units are regarded as a virtual layer having 0 layers. A hidden layer is neither an input or output layers, that is Fig. 4, has 1 hidden layer and 1 output layer and its shows all the connections between the units in the layers. It is clear that a layer only connects to the previous layer. FFNN applications is classified into two such as control of dynamical systems [32, 33], and spaces where the classic machine learning techniques are applied [34]. NNs with two or more hidden layers are called deep networks because the network has become complex with more than 1 hidden layer. Unlike FFNN, the feed-backward neural network (FBNN) can use internal state “memory” (store information) to process sequence of data inputs. That means FFNN can logically handle task according to first come first serve bases of inputs.

**Fig. 5:**
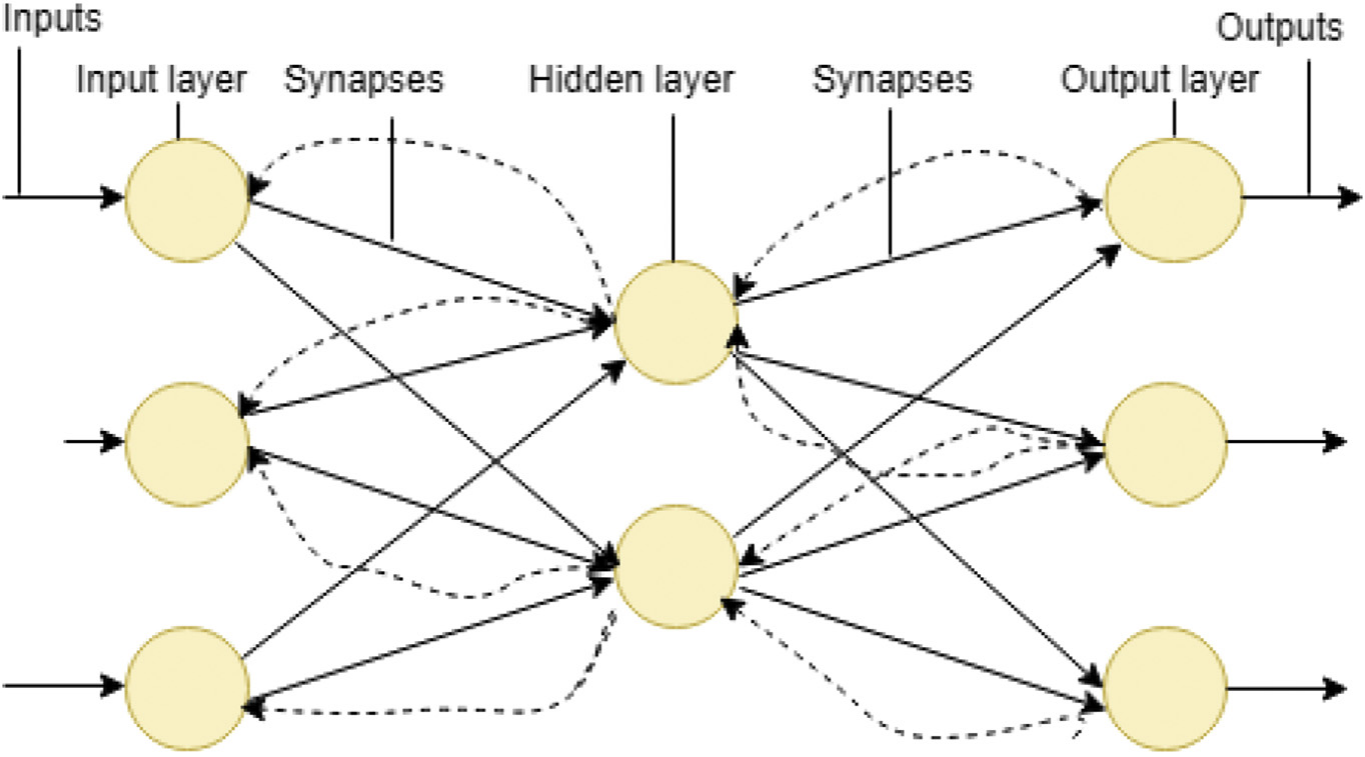
Feed-backward neural network.

Feed-backward NN can applied to tasks like un-segmentation, and pattern recognition (connected handwriting recognition). Feed-backward neural network application areas include mathematical proofs, seismic data fitting, medicine, science, engineering, classification, function estimation, and time-series prediction, etc. An architecture of FBNN illustrated as in Fig. 5.

In feedback NNs or backpropagation, connections between nodes produced a coordinated graph in sequence. The coordinated graph in sequence allows feedback NNs to demonstrate dynamic terrestrial behaviour for a time sequence. Examples are Kohonen's self organizing map and recurrent neural network (RNN). RNN referred to a standard kind of neural network which extended over time, with edges that feed into the next time step rather than feeding into the next layer concurrent time of step. RNN is constructed to sequences recognition, for instance, a text or a speech signal. It has cycles within that indicates presence of short-memory in the net. Unlike a recurrent neural network, an RNN is like a hierarchical network where the input need processing hierarchically in the form of a tree because there is no time to the input sequence.

### 2.4 Deep learning

**Fig. 6:**
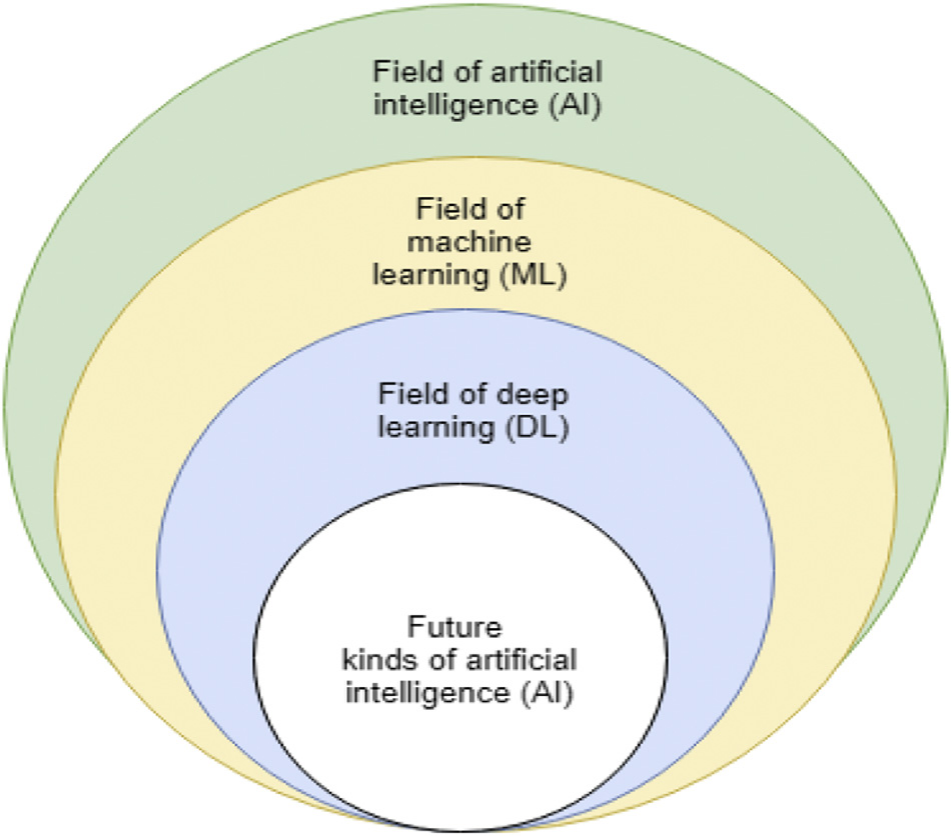
Artificial intelligence development and expansion.

Artificial intelligence (AI) has existed over many decades, and the field is wide. AI can be view as a set that contains machine learning (ML), and deep learning (DL). The ML is a subset of AI, meanwhile, DL, in turn, a subset of ML. That is DL is an aspect of AI; the term deep learning refers to artificial neural networks (ANN) with complex multilayers [35]. The distinction between deep learning and neural networks like feedforward NNs and feed backward NNs lies in their characteristic. Deep learning has more complex ways of connecting layers, also has more neurons count than previous networks to express complex models, more also with more computing power to train and further has automatic extraction of the feature. Therefore, DL defined as a NN with a broad variables and layers with a single basic network architecture of unsupervised pre-trained networks, convolutional NNs, recursive NNs, and recurrent NNs. The technological development in the field of AI has expanded over time as showed in Fig. 6.

DL methods have been found to be fitting for big data study with remarkable success in its applied to speech recognition, computer vision, pattern recognition, recommendation systems, and natural language processing [36]. Nowadays, the innovation of DL in image identification, object detection, image classification, and face identification tasks have great success. The research by Zhou et al. [37] presents an application of DL in object detection task and its speedy use in the domain of computer vision. Similarly, the recently reviewed work by Liu et al. [36] discusses popularity of DL architectures and their industrial and practical applications. The review provided a comprehensive knowledge on four DL architectures like, deep belief network, restricted Boltzmann machine, autoencoder, and convolutional neural network (CNN).

One of the most common deep NNs is the convolutional NN called CNN. A CNN is a standard NN that extends across space via shared weights. CNN is designed to recognize images by having convolutions within, that can recognize the image of an object. CNN has multiple layers; including fully-connected layer, pooling layer, convolutional and non-linearity layers. The fully connected layers and convolutional layers have parameters, however non-linearity layers and pooling do not have parameters. Study have shown that CNN has an excellent performance in ML problems [35]. Particularly, in the applications to image data, like the most extensive image classification dataset, natural language processing, and computer vision.

The major concept of deep learning (DL) is learning data representations by increasing the quality of handling the ideas rather than events (abstraction) levels. Mostly in all levels, a significant amount of quality ideas or abstraction representation at a advance level are known through definition regarding fewer quality ideas or non-representations at the basic levels. This type of stages of learning, growth or hierarchical process of learning is superb because it can enable a system to fathom complex or multi-complex presentations accurately from raw data [38, 39]. This superb characteristic is making deep learning applicable to different fields.

To fast tract classification, recognition of patterns in biological data, many methods of artificial intelligence particularly the machine learning has been proposed [38, 40, 41]. Artificial intelligence (AI) is a combination of reinforcement learning (RL) and deep learning (DL), thus mathematically, AI = RL + DL. Machine learning has become the latest model to digital evolution, that is making computing processes more cost-effective, efficient, strong, dependable and reliable.

Conventional machine learning techniques widely classified into two sets that is, supervised and unsupervised. The supervised learning has the capability of classifying objects in a pool with possibility of given features or attributes nor annotations. A typical example of supervised learning is when students have written an exam, and having the exam marked by the teacher and shown which questions the students answered incorrectly. After being shown the correct answers, the students are expected to learn then understand how to answer those questions correctly.

But the unsupervised learning methods form clusters or groups between and among the objects in an area to identify likeness, then use similarity for classifying unknowns. Example of unsupervised learning is the man that is learning how to ride a car by himself. He will start by entering the car and start the engine with the ignition key, then put down the clutch and put the car in first gear and press the accelerator for the car to move forward and then manage to control the steering. He continually practices the driving steps in a large open field and gradually master the driving technique, and then over time start to enter the road to drive skillfully.

The reinforcement learning (RL) category, enables a system or an agent learn from the previous experiences gains in the environment through interaction and observing the results of these interactions. The interaction helps to mimic or imitates the basic pattern in which humans and animals learn. An agent of RL can act, and each act influences the agent's future condition, a scalar reward signal measures RL success. RL goal is to choose actions that maximize future gain. In nutshell, DL, is a general-purpose framework in making decision. A framework that present learning given require objective from raw inputs by applying minimal domain knowledge. For instance, RL completely bypasses the problem of rules learning in a task. The learning agent learns by participating in the given activity.

An example is a game of Chessboard, to determine the best action play move, the players need to think about various possibilities and strategies. The amount of possibilities is potentially big that it is not possible to perform a brute-force search. However, if a machine is to be built to play such a game using traditional techniques, there will be a necessity to specify many rules to cover all these possibilities. Reinforcement learning completely bypasses this problem. Examples of RL include value based deep RL, model based deep RL and policy based deep RL.

While common supervised artificial neural networks and their adaptations include single-layer perceptron (SLP) [10, 31], multilayer perceptron (MLP) [42, 43], and linear classifiers [44, 45]. Also, popular supervised ANNs include support vector machines (SVMs) [46, 47], k-nearest neighbours (kNNs) [48]. More also in the category of popular supervised ANNs are Bayesian statistics [49], decision trees [50] and hidden Markov model (HMM) [51]. Meanwhile, some common unsupervised methods include k-means [52], expectation maximization [53], and autoencoders [54]. Other unsupervised approaches in literature are density-based [55], self-organizing maps [56], clustering [57] and fuzzy [58].

Kernel-based neural networks methods are a class of algorithms for pattern analysis, like SVM (support vector machine). Kernel functions or algorithms used in shallow architectures, for example support vector machines (SVMs), or multilayer kernel machines (MKMs). An investigation study by Cho and Saul [59], evaluates MKMs and SVMs with kernel functions to demonstrate advantages of deep NN architectures. The study highlighted the usefulness of kernel-based neural networks in the applications to optical character recognition and DNA analysis. The research by Camps-Valls, and Bruzzone [60], proposed kernel-based techniques for classification of hyperspectral image, with main characteristics of various kernel-based methods. The result demonstrates the successful performance of standard support vector machines (SVMs) that regularized radial basis function NNs (Reg-RBFNN), AdaBoost (Reg-AB) and kernel Fisher discriminant (KFD) analysis.

ANNs has significant advantages over statistical models when both are relatively compared. In ANN models there are no assumptions about data properties or data distribution. Therefore, ANNs are more useful in practical application. Also, unlike some statistical models that require certain hypothesis for testing, ANN models do not require any hypothesis. ANNs are very flexible, data reduction models, encompassing nonlinear regression models, and discriminant models. More also, unlike the support vector machine, extreme learning machine, and random forest, ANNs are more fault tolerant. That is, they can handle incomplete data and noise, they can solve non-linear problems, Also, trained ANNs, can generalize at high speed and make predictions. Furthermore, ANNs are scalable when relatively compared to the support vector machine, extreme learning machine, and random forest.

Interestingly, DL techniques attracted more research attention since year 2006. DL has the built-in ability to solve the defect of traditional paradigms dependent on hand-built materials. Also, DL approaches have been useful in big data technique with performance applications to pattern recognition, natural language processing, recommendation systems, speech recognition, and computer vision.

### 2.5 Modeling

Generally, modeling is the process of presenting a real-world phenomenon or object as a set of mathematical expression. However, neural network modeling is a process representing the way the nervous system functions. That is, a NN is a simplified representation of how the man brain processes information. Its function by simulating many interconnected processing units that resemble idea versions of neurons. It is the most common optimization paradigm used in optimizing a neural network. Recently gradient descent used in updating weights in a NN model, that is updating and changing the model's parameters in a direction to minimize the Loss function.

A study by Ludermir, Yamazaki, and Zanchettin [61], proposed a new method for NN global optimization. The method combines backpropagation algorithm, annealing, and tabu search, to produce an automation for generating networks with low complexity and high classification. The results showed that the new method is better than the previous studies obtained by the most used optimization approaches. Recently, researchers synthesized artificial enzymes that functions or operates in the metabolism of living cells. These artificial enzymes used cell's own energy that enable hydrogen gas production from solar energy [62]. ANNs have applied in many ways like in system control [63], pattern recognition [64], power systems [65], robotics control [66], forecasting [67], manufacturing [68], social sciences [69], Art [70], optimization [71], psychological sciences [72], signal processing [73] etc.

In an optimizer modeling approach to a solution of reusability problem. An example of a practical approach to modeling in the optimizer for a software architecture. A published work by Delinchant et al. [74] discusses an optimization framework in software components paradigm for sizing. Delinchant et al. work presents a framework system design based on optimization. It then highlights, a generator (analytical expressions of the framework system), an optimization service, the component standard and the pattern of using the designed framework. The result demonstrates how the framework system for the software components can be used in building new generations that optimized environment. That allow capitalization and reutilization by the combined software packages and optimization algorithms. Thus, the approach showed the possibility of building a global software architecture that optimized systems and components.

In measuring a good prediction model to predict the expected outcome, a loss function required. That is the group or class of functions that minimized is called “loss functions”. A most popular used method of finding the minimal point of a function referred to as gradient descent. DNNs are currently among the most popularly used classifiers. In modeling on loss function, a paper by Janocha, and Czarnecki [75], investigated the effect of loss functions choices on deep models and its learning dynamics, and the resulting classifiers robustness to different effects. Two experiments (L1 and L2) performed on classical datasets. The result shows that L1 and L2 losses are, quite interesting, that justified classification objectives for deep neural nets because it gives probabilistic interpretation regarding expected misclassification.

In finding the optimum design that provides a lightweight and high quality at the same time, it is significant to have effective and efficient prediction methods at the initial design stage. In a research work on weight optimization method, the paper by Yu, and Chi [76], proposed a weight model optimization in credit evaluation, based on the concept that the optimal is the weight. That is, after empowerment the result of credit evaluation should has maximum discriminating power to differentiate non-default from default customers. The empirical results demonstrate that the discriminating power of credit evaluation was the strongest compared with the three types of weight models, like t-value, mean square error, and variation coefficient.

### 2.6 ANN emerging successes and applications

In the recent times various successful used of ANNs emerged in catalysis, meteorology, biology, chemistry, physics, nuclear physics, high-energy physics, and other areas of science. Nowadays, ANN has found uses in a new area such as in catalyzing especially in the chemical industrial sector. Catalysis is term as the significant energy in the modernization process of chemical industries. It ensures effective, efficient and successful use of finite natural resources, it prevent waste and air pollution, and provides safety for the industrial sector. Catalysis become the foundation of large scale operations regarding size in chemistry and petrochemistry environment. However, as demand changes, new environmental challenges now require new catalytic solutions. For example, changed in the energy economy has driven an increasing demand for coal and gas, hence given room for new challenges for catalytic technology in the areas like liquefaction in material science [77, 78].

Recently, there have been reported cases of ANNs applications to catalysis research in the literature. The reviewed paper by Li, Zhang, and Liu [79], show how ANNs applied to catalysis helps people in addressing the complex problems and then accelerating the progress of the catalysis utilization. The reviewed paper further showed how ANNs applied in many ways to catalysis prediction, new catalysts design, and understanding of catalytic structures which produced effective result. Likewise, research by Corma et al. [80] demonstrates how artificial NNs applied to modeling catalytic data in combinatorial catalysis and to predicting a new catalyst composition for ODHE (oxidative dehydrogenation of ethane).

In another development research on an accurate description of chemical processes using computational methods like density functional theory (DFT). Behler and Parrinello [81], introduces a new breed of NN model of DFT, that gives the energy as a function of all atomic positions in systems with arbitrary size and in various orders of magnitude that is faster than DFT. The high level of accuracy of the NN method is shown for bulk silicon compared with DFT. This NN approach is generic which can be apply to all kind of periodic and nonperiodic systems.

Recently, a generalized ML input representation was applied by quantification and concentrations of blended solutions in addressing the problem of determining intrinsic trends in CO_2_ solubility under a specific condition. The research orchestrated by Li, and Zhang [82], applied general regression NN (GRNN) algorithm in fitting intrinsic trends or movement of CO_2_ solubility with a minimal amount of experimental data. Which resulted in the average RMSE (root mean square error) less than 0.038 mol CO_2_/mol of solution. The study has shown that applied generalized input representation, could provide a better comprehension of the inherent trends of CO_2_ solubility in a blended amount of solutions.

ANNs are useful and applicable in system modeling like in implementing system identification and complex mappings. For instance, the application of NNs to renewable energy challenges have shown tremendous success. In 2001, an investigation by Kalogirou [83], applied ANN to energy like solar regarding modeling and design of a solar steam generating plant. The experimental result demonstrates prediction for speed, load, and error.

Wavelet networks are been used extensively and effectively in different engineering fields for classification, identification and control problems. Wavelet networks are feed forward networks that uses wavelets as activation functions. ANNs application to solar radiation data forecasting with adaptive wavelet network has been useful. The most recent work by Li, and Liu [84], uses an adaptive wavelet network architecture in discovering an appropriate model for forecasting the daily total amount of solar radiation. The daily total amount of solar radiation is considered the most significant in the prediction of the performance of renewable energy like solar, importantly in sizing photovoltaic (PV) phenomenon of power systems. That is in the conversion of light into electricity. The experimental results demonstrate that the ANN model predicts daily total amount of solar radiation parameters with an accuracy of 97% with a mean absolute percentage error of 6%. Also, Li, and Liu proposed a model on the optimization of the solar water heater and performance prediction using a knowledge-based machine learning technique. The result demonstrates that the model generalization that can applied in different locations even for weather data, like ambient temperature and sunshine period.

In recent years ANN application to chemistry and physics problems has increasingly popular and successes [85]. Many applications of approximation techniques and standard approaches to data fitting are performing better in NN. NN provide more accuracy with a lower number of adjustable parameters than any other methods. Learning in NNs is understood when it rebuilds hypersurfaces together with a sample points, generalization, and interpolation. NNs apply sigmoidal functions to re-buildings or transformations, stated in most physics and chemistry problems. Thus, resolving an arbitrary data fitting issue by applying a single layer net architecture if there is no restriction in sigmoidal functions applied [85].

Neural networks application in physics has witnessed a remarkable success. The research by Lynch et al. [86], describes new opportunities for applying NNs to physics and mapping kinds of physics related problems, especially in the field of science and engineering problem. ANN has been found useful in predicting the concentrations of radioactivity. An understanding the levels of radioactivity values with values of other variables in the environment can be apply to train a network to estimate the next levels of radioactivity. Hence, accuracy of the NN method can be better than other approaches in certain monitoring areas.

Nuclear theory main goal is to predict nuclear structure and nuclear reactions from the fundamental theory of high cohesion interactions, and quantum chromodynamics (QCD). With the current superb high-performance computing (HPC) systems, many ab initio methods, like the No-Core Shell Model (NCSM), have been designed to calculate the chemical properties of atomic nuclei. However, to accurately calculate the properties of atomic nuclei, there are a plethora of theoretical and computational challenges involves. To overcome this problem, a most recent study on atomic nuclei properties prediction by Negoita et al. [87] proposes a feed-forward ANN for predicting the properties of atomic nuclei such as ground state (gs) energy and ground state (gs) point proton root-mean-square (rms) radius. The result demonstrates that FFANN could predict properties of the ^6^Li nucleus like the gs energy and the gs point proton rms radius. The result satisfied the ideal physics condition. An important advantage of the ANN method is that it does not require mathematical relations of the input data and output data. Importantly, ANN applications to Physics and Chemistry problems should be compared with other methods like statistical techniques and data fittings procedures for the performance measure.

### 2.7 ANN models in different application areas

Many artificial neural network techniques have been adopted in the academia and industries to address the challenges in computer vision, speech and pattern recognitions, face alignment, and detection. These include;

#### 2.7.1 Speech recognition

The application of ANNs has become divergence and understood in the capability of its successes in speech or communication recognition. In the past decades, ML algorithms have applied widely in areas like acoustic modeling and ASR (automatic speech recognition) [88].

#### 2.7.2 Computer vision

Computer vision aims at making computers to accurately understand and process visual data efficiently like videos [88] and images [89, 90, 91]. Main goal of computer vision is to provide computers with the kind of ability of man brain functionality. Theoretically, computer vision alludes to the logical control which studies how to separate data from images in artificial frameworks. Sub domains of computer vision include object detection and object recognition, object estimation, object position, event detection, scene reconstruction, image restoration, image editing, video enhancement, and statistical learning. Hence, in computer vision, ANN models are very useful.

#### 2.7.3 Pattern recognition

The recent improvement in deep learning models has given novel ways to deal with the issue in recognition of a pattern or pattern recognition (PR). PR is a scientific area that focus in identification of sequence in each input [92, 93]. PR is a general concept that surrounds various subdomains such as speech tagging, regression, sequence labeling and classification. There are rapidly increasing needs for information processing and output, due to industrial development, that has new trend and challenges to PR.

#### 2.7.4 Face alignment

Face alignment plays a role that is significant in diverse visual applications. In recent times ANNs has claimed successes in face alignment [94, 95, 96] and face recognition [97, 98, 99] and other models [100] have shown successes. Interestingly DL techniques can be applying to explain genetic variants to identify pathogenic variants [101, 102]. Usually, combined annotation dependent depletion algorithm is popularly applied to interpret the coding and non-coding variants.

#### 2.7.5 Detection

Detection in medical diagnosis, security, image objects, financial irregularity, a fault in a system, are being enhanced through ANNs application. Thus, ANN plays an essential role in the detection, particularly when applied to breast cancer [103, 104, 105, 106]. The performance of ANN can be relatively compared with other approaches in crime detection such as DNA and activity profiling [107] and the use of big data for financial crime detection [108]. Despite the many publications in the utilization of NN in different medical challenges, but there are few reviews study available that explain the architecture in improving the detection methods regarding performance, accuracy, sensitivity, and specificity. Thus, detection capability is commonly known subdomain or computing in computer vision which seeks to understand, locate, classify or differentiate the targeted image objects. An example during detection tasks, an image can be scanned to know certain special features or characteristics. For instance, using of image detection in medical diagnosis, especially abnormal cells or tissues in medical images.

Normally, traditional methods are base on hand-designed features and contrast inference mechanisms. The DL techniques require raw image data only [109, 110, 111, 112]. Also, DL techniques applied to Glaucoma detection with promising results [113, 114, 115]. More also, ANN has employed in image change and computer vision detection in both civil and military challenges. Recently, image detection applying in remote sensing, disaster evaluation, videoing and surveillance. Furthermore, ANNs or deep learning techniques has been applying to human-robot interaction systems that yielded results [116, 117, 118].

### 2.8 Comparison of different ANN models

This section highlights discusses, compares, summarizes and critiques more than eighty research articles on artificial neural network model's application to the diverse area of the economy. The comparison was made based on (i) author(s)/year of publication (ii) ANN modeling (iii) ANN area of application (iii) studied contribution to human challenges (with references to Supplementary Table 2) [11, 119, 120, 121, 122, 123, 124, 125, 126, 127, 128, 129, 130, 131, 132, 133, 134, 135, 136, 137, 138, 139, 140, 141, 142, 143, 144, 145, 146, 147, 148, 149, 150, 151, 152, 153, 154, 155, 156, 157, 158, 159, 160, 161, 162, 163, 164, 165, 166, 167, 168, 169, 170, 171, 172, 173, 174, 175, 176, 177, 178, 179, 180, 181, 182, 183, 184, 185, 186, 187, 188, 189, 190, 191, 192, 193, 194].

#### 2.8.1 Summary and critiques

The comprehensive review has shown that interest in ANN applications exploded over the past two decades. The results indicate that the selected research articles are recent because they were majorly published between the year 2009–2018 and focuses on developmental and technological issues regarding ANN. The survey covers various areas of ANN applications such as computer security, network security, science and engineering, medical science, biology, ecology, nuclear industry, electricity generation, management, mineral exploration.

Other areas of application are; crude oil fractions quality prediction, crops, water treatment, policy and businesses like banking, insurance, the stock market, money laundering and other financial institutions for crime detection. The field of application varies from science, engineering, socio science, humanity and to art-related fields. From the survey of various articles, it became clear that the artificial neural network application has no boundary. ANNs has attracted the most attention from researchers in recent time, for instance, the study found that forecasting of crops and animals yield are helpful in agricultural development.

Interestingly, this application of ANN has led to an increase in agricultural production which has enhanced food security in many nations. In the past two decades, ANNs have applied in different fields of agricultural, particularly to crop area estimation and classification. Also, agricultural data can be predicted using a neural network. There are diverse approaches proposed for data analysis such as neural network models; examples are; feedforward artificial neural network, back propagation neural networks, probabilistic neural network. Others include supervised associating networks, multi-layer perceptron neural network architectures, learning vector quantization, and multi-layer neural network.

Although, the study by Kitchens, and Harris [195], on ANN application to detection of fraud in insurance business and finance, demonstrates fraud detection process efficiently. However, the result needs comparison with other approaches like data mining, regression model and other statistical models for evidence of successes.

Though, the research by Fanning and Cogger [149], on the application of ANN for fraud detection in management show evidence of success in fraudulent detection using ANN in financial statements. Nevertheless, the result needs to be compared with other current techniques if it performed better or outperformed state-of-the-art detection techniques. More also, even though ANN application is quite novel, one must not ignore that nowadays there are other novels criminal detection techniques with graphical and programming applications like graph-theoretic anomaly detection and inductive logic programming.

Although, ANN tools make it simple, easy and faster for data analysis to enable not only the discovery of a new method for businesses, industrial, and educational topologies but also cross-product, cross-channel and cross-customer performance. Nevertheless, ANN is not necessarily a panacea; there has to be information and communication technology (ICT) involvement in making the data sources available and, of course, ANN tools do not exclude the need for data cleansing. But it gives new opportunities to an organization looking to tackle management challenges such as improvement of materials, product, services, financial crime, and so on via new means.

With emerging technologies such as data mining, genetic algorithms, hybrid models, mathematical models, big data application especially in crime detection and prevention [185, 196]. The evolution of computer and the internet has the influence of rapid technology and digital media growth on every aspect of human lives [197, 198]. In addition to extensive interactive software application [199]. Researchers can follow adequate artificial intelligence application mechanisms to achieve huge success in the diverse field of endeavours. In nowadays of global computing, there are a plethora of benefits in NNs. Since capability to learn during and after training makes ANN very powerful and flexible. Also, ANN do not require paradigm before performing a specific task, that is, without the need in understanding the external or internal mechanisms of the task before implementation [200].

#### 2.8.2 The result of ANN application

The result of ANNs application to different areas of lives and disciplines as found in the literature is presents in Table 1.

**Table 1:** Summarized result on ANNs application regarding prediction, pattern recognition and Classification.

Example of many fields of applications of ANNs	Prediction	Pattern recognition	Classification	Total
Security	20	18	2	40
Science	25	25	2	52
Engineering	22	7	2	31
Medical science	10	5	2	17
Agriculture	3	3	2	7
Finance	10	15	2	27
Bank	5	15	2	22
Weather and climate	2	15	2	19
Education	30	15	2	47
Environmental	10	15	2	27
Energy	5	15	2	22
Mining	2	15	2	19
Policy	2	2	2	6
Insurance	5	4	2	11
Marketing	5	5	2	12
Management	40	2	2	44
Manufacturing	12	15	5	32
Other fields	52	11	10	71

**Fig. 7:**
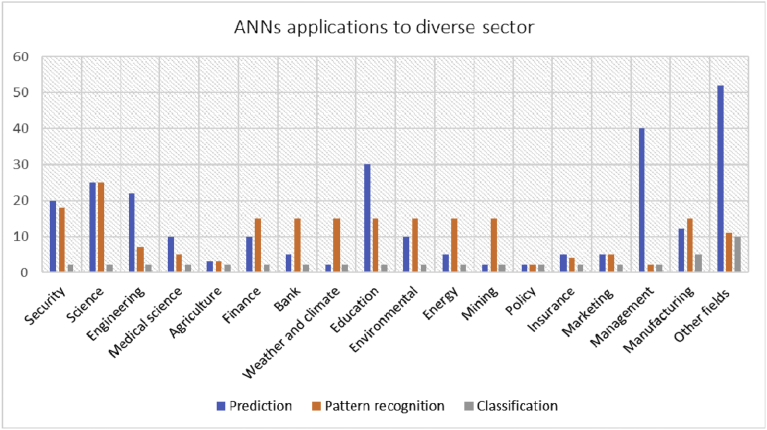
Reviewed ANN applications framework.

Table 1 indicates that ANN models are useful in classification, pattern recognition, claustering, optimisation and prediction. The relationships among many areas of ANN applications further presented in Fig. 7.

The correlation among the distinct fields further reveals that ANN can apply to any areas of studies, industries, and profession. The histogram reveals the areas of application of ANN in security, science, engineering, medical science, agriculture, finance, banking, weather and climate, education, environmental, energy, mining, insurance, marketing etc. Therefore, interested researchers can explore the ANN application in these areas or many other emerging areas for future research for better solution to problems in their fields. Since there is always an algorithm, model, scheme, and framework for any problem.

## 3 Conclusions

The survey was comprehensive with a discussion on how NN could applied to address human needs. ANNs has many names as found in the literature such as; connectionism/connectivist models, adaptive systems, parallel distributed processing models, self-organizing systems, neuromorphic and neurocomputing systems [201, 202, 203, 204].

The ANNs application areas considered in the survey include; computer security, medical science, business, finance, bank, insurance, the stock market, electricity generation, management, nuclear industry, mineral exploration, mining, crude oil fractions quality prediction, crops yield prediction, water treatment, and policy. It is interesting to know that neural network data analysis adds accuracy, processing speed, fault tolerance, latency, performance, volume, and scalability. Many new and enhanced data management and data analysis approaches help in the management of ANN. Creating analytics from the available data that aid in largely prioritizing information and provide its human business value. The ANN analytics in turn help in combating challenges and mitigate any possible risks.

Therefore, based on data analysis factors such as accuracy, processing speed, latency, performance, fault tolerance, volume, and scalability, an evaluation was made of the ANN techniques. Then, proposes that neural-networks models such as FFBP and hybrid model using neural networks are performing better for implementation of human problems when compared to other approaches currently in practice. Also, the study proposes hybrid neural networks models and genetic algorithms (GA) for a better performance regarding effectiveness and efficiency.

ANN are new computational model with rapid and large uses for handling various complex real world issues. ANNs popularity lies in information processing characteristics to learning power, high parallelism, fault tolerance, nonlinearity, noise tolerance, and capabilities of generalization.

### 3.1 Suggestions

Based on the reviewed literature, some areas of improvement can be suggested to professionals, researchers and newcomer researchers for further research and future research development. The BPNNs and FFPNNs have the potential for modeling variables. In optimizing BPNNs and FFPNNs performance, a systematic approach is requiring in the model development process. Therefore, the following suggestions are stated.

1. Transformation of data. The past and current studies indicate that it is unnecessary to transform or change data not normally or usually distributed that reveal non-regular periodic development or variant. Meanwhile, the modification of heteroscedasticity and trends in data are encourage. Also, data normalization and scale to commensurates with function transfer in output layer.
2. Determining of appropriate inputs model. Input variables determined with the support of a priori knowledge, using a stepwise model-building method or analytical method like cross-correlation technique.
3. Network geometry choice. One hidden layer may be enough as adequate in most network practical uses. However, mathematical expression could determined the upper bound of hidden layer nodes required to approximate continuous function. Except if nonconvergent techniques, like cross-validation, the connection between the quantity of training and the quantity of hidden layer units likewise should be considered. The relationship can be investigated with the guide of the rules given in some literature.
4. Researchers can focus on network characteristic at variable specification phase. Also, it is useful to conduct trials in determining required local minimum in the error surface, and oscillations in the R.
5. Forecasting with continuous training, with different step sizes taken in weight space. These steps can be useful when selecting appropriate network parameters like (transfer function, momentum, epoch size, learning rate, and error) and how many training samples in the network for a case study.
6. Validation of model. The validation of model is necessary for standardization and practical scenarios of ANNs for optimization of performance.

### 3.2 Future directions

This research has many areas in need of further investigation. Further research is requiring in the following areas:

(i) A greater focus on adaptive dynamic programming (ADP) could produce interesting findings that account more for significant contributions in the area of brain research and computational intelligence
(ii) Emerging topic in engineering and computer science include parameter adjustment technique in machine learning algorithms. The successful use of algorithms optimization in adjusting network variables should be well studied.
(iii) Further investigation into the applications of reinforcement-learning, unsupervised, semi-supervised methods to the deep neural network for complex and multi-complex systems.
(iv) Future research should concentrate on the use of intelligent analysis such as neural network models, backpropagation neural networks, probabilistic neural network, supervised associating networks, multi-layer perceptron neural network architectures, learning vector quantization, multi-layer neural network, and hybrid neural network models. These are because the areas can provide better performance in the application of neural network to diverse challenges of life.
(v) Since the use of inferential statistics and neural networks can be more predictive in data analysis. Hence, the predictive approach can also be another focus in the study of the subject.
(vi) Research is requiring in the execution of DL algorithms for communication materials like mobile gadgets. As recently, DL chips idea emerged that is attracting research interest.
(vii) Public awareness training on artificial network neural models should continue regularly, especially on the need for providing useful information using empirical analysis and digital data.
(viii) More investigation is requiring in the stability analysis of deep NN because, in recent times, the DNNs stability analysis has become a hot topic for research focus due to its advantages in many industrial sectors.
(ix) With the new trend in big data technique, DL would be useful where large amounts of un-supervised data are applied. It would be interesting to construct DL models that can learn from fewer training data, particularly for visual and speech recognition systems.
(x) Further research should be a focus on the use of DNNs to nonlinear networked control systems. More understanding of complicated dynamics would help us establish how to obtain better performances in control, and filtering capability effectively and efficiently.
(xi) The governments and institutions need to provide funding into diverse areas of application of neural networks for success, especially in this era of modern education, technology advancement, industrial growth, economic challenges, artificial intelligence development, and information and communication revolution.

### 3.3 Other areas of further research

It would be a good idea for further research to be carried out in the following areas:

(i) Genetic algorithms (GA) for better performance regarding effectiveness and efficiency.
(ii) Brain research focusing on RL and ADP (adaptive dynamic programming) and RL to produce more success in performing intelligent optimization.
(iii) Hybrid neural networks models for better performance regarding effectiveness and efficiency.
(iv) Research into possibilities in the integration of neural networks with other existing or developing technologies.
(vi) There is a need for more future research into the application of ANN technology in businesses, industries, energy and agriculture for development and wealth creation.
(vii) Research focus on determining if the more profitable trading system can be implemented using hybrid intelligent systems, expert systems, genetic algorithms, combining fuzzy logic and ANNs.
(viii) Research to explore the possibility of using ANNs to forecast time series, and the integration of expert systems and fuzzy logic in determining trading signals.
(ix) An exploration into the possibility of using genetic algorithms to select input variables and optimal parameters for a system.

Meanwhile, the research on ANNs applications will attain more significant progress in the nearest future.

## Declarations

### Author contribution statement

All authors listed have significantly contributed to the development and the writing of this article.

### Funding statement

This research was partially supported by two organizations. Center for Cyber Safety and Education, United States Internal Revenue segregated fund of (ISC)², Inc. Code. EIN: 45-2405127 through the (ISC)2 graduate cybersecurity scholarship award, 311 Park Place Blvd. Suite 610 Clearwater, FL 33759 USA. Also, by Fundamental Research Grant Scheme (FRGS) for ‘‘content-based analysis framework for better email forensic and cyber investigation” [203/PKOMP/6711426], SFRG Lab, School of Computer Sciences, Universiti Sains Malaysia, Penang, 11800, Malaysia.

### Competing interest statement

The authors declare no conflict of interest.

### Additional information

No additional information is available for this paper.

## References

[1] Dave V.S., Dutta K. (2014). Neural network-based models for software effort estimation: a review. Artif. Intell. Rev..

[2] He H., Garcia E.A. (2009). Learning from imbalanced data. IEEE Trans. Knowl. Data Eng..

[3] Mozaffari A., Emami M., Fathi A. (2018). A comprehensive investigation into the performance, robustness, scalability and convergence of chaos-enhanced evolutionary algorithms with boundary constraints. Artif. Intell. Rev..

[4] Izeboudjen N., Larbes C., Farah A. (2014). A new classification approach for neural networks hardware: from standards chips to embedded systems on chip. Artif. Intell. Rev..

[5] Wang D., He H., Liu D. (2018). Intelligent optimal control with critic learning for a nonlinear overhead crane system. IEEE Transact. Ind. Inf..

[6] Xing F.Z., Cambria E., Welsch R.E. (2018). Natural language based financial forecasting: a survey. Artif. Intell. Rev..

[7] Kingston G.B., Maier H.R., Lambert M.F. (2005). Calibration and validation of neural networks to ensure physically plausible hydrological modeling. J. Hydrol..

[8] Flood I., Kartam N. (1994). Neural networks in civil engineering. Principles and understanding. J. Comput. Civ. Eng..

[9] Minns A.W., Hall M.J. (1996). Artificial neural networks as rainfall-runoff models. Hydrol. Sci. J..

[10] Tokar A.S., Johnson P.A. (1999). Rainfall-runoff modeling using artificial neural networks. J. Hydrol. Eng..

[11] Boyacioglu M.A., Kara Y., Baykan Ö.K. (March 2009). A predicting bank financial failures using neural networks, support vector machines and multivariate statistical methods: a comparative analysis in the sample of savings deposit insurance fund (SDIF) transferred banks in Turkey. Expert Syst. Appl..

[12] Haykin S., Network N. (2004). A comprehensive foundation. Neural Network..

[13] Rahmanifard H., Plaksina T. (2018). Application of artificial intelligence techniques in the petroleum industry: a review. Artif. Intell. Rev..

[14] Araque O., Corcuera-Platas I., Sanchez-Rada J.F., Iglesias C.A. (2017). Enhancing deep learning sentiment analysis with ensemble techniques in social applications. Expert Syst. Appl..

[15] Jahnavi M. (Jul 9, 2017). Introduction to Neural Networks, Advantages and Applications, towards Data Science. http://www.deeplearningtrack.com.

[16] Wang D., He H., Liu D. (2017). Adaptive critic nonlinear robust control: a survey. IEEE Trans. Cybern..

[17] Haykin S. (2009). Neural Networks and Learning Machines.

[18] Haykin S. (1996). Neural networks expand SP's horizons. IEEE Signal Process. Mag..

[19] Stergiou C., Siganos D. (1996). Neural Networks 1996.

[20] Huang T.J. (2017). Imitating the brain with neurocomputer a “new” way towards artificial general intelligence. Int. J. Autom. Comput..

[21] Haykin S.S. (2001). Kalman Filtering and Neural Networks.

[22] Yu W., He H., Zhang N. (2009). Advances in Neural Networks, ISNN 2009 6th International Symposium.

[23] Fan W., Bouguila N., Ziou D. (2012). Variational learning for finite Dirichlet mixture models and applications. IEEE Trans. Neural Network. Learn. Syst..

[24] Saravanan K., Sasithra S. (2014). Review on classification based on artificial neural networks. Int. J. Ambient Syst. Appl. (IJASA).

[25] Martínez-Porchas M., Villalpando-Canchola E., Vargas-Albores F. (2016). Significant loss of sensitivity and specificity in the taxonomic classification occurs when short 16S rRNA gene sequences are used. Heliyon.

[26] Abid F., Hamami L. (2018). A survey of neural network based automated systems for human chromosome classification. Artif. Intell. Rev..

[27] Das K., Behera R.N. (2017). A survey on machine learning: concept, algorithms and applications. Int. J. Innovat. Res. Comput. Commun. Eng..

[28] Boutaba R., Salahuddin M.A., Limam N., Ayoubi S., Shahriar N., Estrada-Solano F., Caicedo O.M. (2018). A comprehensive survey on machine learning for networking: evolution, applications and research opportunities. J. Internet Serv. Appl..

[29] Ogwueleka F.N., Misra S., Colomo-Palacios R., Fernandez L. (2015). Neural network and classification approach in identifying customer behavior in the banking sector: a case study of an international bank. Hum. Factors Ergon. Manuf. Serv. Ind..

[30] Hagan M.T., Menhaj M.B. (1994). Training feedforward networks with the Marquardt algorithm. IEEE Trans. Neural Network..

[31] Hertz J.A. (2018). Introduction to the Theory of Neural Computation.

[32] Kuschewski J.G., Hui S., Zak S.H. (1993). Application of feedforward neural networks to dynamical system identification and control. IEEE Trans. Contr. Syst. Technol..

[33] Xiang K., Li B.N., Zhang L., Pang M., Wang M., Li X. (2016). Regularized Taylor echo state networks for predictive control of partially observed systems. IEEE Access.

[34] De Martino A., De Martino D. (2018). An introduction to the maximum entropy approach and its application to inference problems in biology. Heliyon.

[35] Albawi S., Mohammed T.A., Al-Zawi S. (2017). Understanding of a convolutional neural network. International Conference on Engineering and Technology (ICET).

[36] Liu W., Wang Z., Liu X., Zeng N., Liu Y., Alsaadi F.E. (2017). A survey of deep neural network architectures and their applications. Neurocomputing.

[37] Zhou X., Gong W., Fu W., Du F. (2017). Application of deep learning in object detection. IEEE/ACIS 16th International Conference on Computer and Information Science (ICIS).

[38] Mahmud M., Kaiser M.S., Hussain A., Vassanelli S. (2018). Applications of deep learning and reinforcement learning to biological data. IEEE Trans. Neural Network. Learn. Syst..

[39] Bengio Y. (2009). Learning deep architectures for AI. Found. Trends Mach. Learn..

[40] Tarca A.L., Carey V.J., Chen X.W., Romero R., Drăghici S. (2007). Machine learning and its applications to biology. PLoS Comput. Biol..

[41] Fern A.S., Delgado-Mata C., Velazquez R. (November, 2001). Training a single-layer perceptron for an approximate edge detection on a digital image. Technologies and Applications of Artificial Intelligence (TAAI), 2011 International Conference on (pp. 189–193).

[42] Anarghya A., Harshith D.N., Rao N., Nayak N.S., Gurumurthy B.M., Abhishek V.N., Patil I.G.S. (2018). Thrust and torque force analysis in the drilling of aramid fibre-reinforced composite laminates using RSM and MLPNN-GA. Heliyon.

[43] Singh G., Sachan M. (2014). Multi-layer perceptron (MLP) neural network technique for offline handwritten Gurmukhi character recognition. IEEE International Conference on Computational Intelligence and Computing Research (ICCIC).

[44] Kolchinsky A., Lourenço A., Li L., Rocha L.M. (2013). Evaluation of linear classifiers on articles containing pharmacokinetic evidence of drug-drug interactions. Biocomputing.

[45] Bywater R.P., Middleton J.N. (2016). Melody discrimination and protein fold classification. Heliyon.

[46] Zhang Y., Deng Q., Liang W., Zou X. (2018). An efficient feature selection strategy based on multiple support vector machine technology with gene expression data. BioMed Res. Int..

[47] Cao J., Fang Z., Qu G., Sun H., Zhang D. (2017). An accurate traffic classification model based on support vector machines. Int. J. Netw. Manag..

[48] Keller J.M., Gray M.R., Givens J.A. (1985). A fuzzy k-nearest neighbor algorithm. IEEE Trans. Syst. Man Cybern..

[49] Cardona T. (2018). Early Archean origin of heterodimeric Photosystem I. Heliyon.

[50] Tharaha S., Rashika K. (2017). Hybrid artificial neural network and decision tree algorithm for disease recognition and prediction in human blood cells. International Conference on Innovations in Information, Embedded and Communication Systems (ICIIECS).

[51] Tang X. (2009). Hybrid Hidden Markov Model and artificial neural network for automatic speech recognition. PACCS'09. Pacific-Asia Conference on Circuits, Communications and Systems.

[52] Koonsanit K., Jaruskulchai C., Eiumnoh A. (2012). Parameter-free K-means clustering algorithm for satellite imagery application. International Conference on Information Science and Applications (ICISA).

[53] Verma N.K., Dwivedi S., Sevakula R.K. (2015). Expectation maximization algorithm made fast for large scale data. IEEE Workshop on Computational Intelligence: Theories, Applications and Future Directions (WCI).

[54] Baldi P. (2012). Autoencoders, unsupervised learning, and deep architectures. Proceedings of ICML Workshop on Unsupervised and Transfer Learning.

[55] Reddy K.S.S., Bindu C.S. (February 2017). A review on density-based clustering algorithms for big data analysis. International Conference on I-SMAC (IoT in Social, Mobile, Analytics and Cloud)(I-SMAC).

[56] Miljković D. (2017). Brief review of self-organizing maps. MIPRO.

[57] Ahalya G., Pandey H.M. (February 2015). Data clustering approaches survey and analysis. International Conference on Futuristic Trends on Computational Analysis and Knowledge Management (ABLAZE).

[58] Wang G., Huang L., Zhang C. (2006). Study of artificial neural network model based on fuzzy clustering. WCICA 2006. The Sixth World Congress on Intelligent Control and Automation (Vol. 1, pp. 2713–2717).

[59] Cho Y., Saul L.K. (2009). Kernel methods for deep learning. Advances in Neural Information Processing Systems.

[60] Camps-Valls G., Bruzzone L. (2005). Kernel-based methods for hyperspectral image classification. IEEE Trans. Geosci. Rem. Sens..

[61] Ludermir T.B., Yamazaki A., Zanchettin C. (2006). An optimization methodology for neural network weights and architectures. IEEE Trans. Neural Network..

[62] Science News (October 4, 2018). Artificial Enzymes Convert Solar Energy into Hydrogen Gas. https://www.sciencedaily.com/releases/2018/10/181004155426.htm.

[63] Demiroren A., Zeynelgil H.L., Sengor N.S. (2001). The application of ANN technique to load-frequency control for three-area power system. IEEE Porto Power Tech Proceedings (Vol. 2, pp. 5-pp).

[64] Ma H., Chan J.C., Saha T.K., Ekanayake C. (2013). Pattern recognition techniques and their applications for automatic classification of artificial partial discharge sources. IEEE Trans. Dielectr. Electr. Insul..

[65] Vankayala V.S.S., Rao N.D. (1993). Artificial neural networks and their applications to power systems—a bibliographical survey. Elec. Power Syst. Res..

[66] Gopalapillai R., Vidhya J., Gupta D., Sudarshan T.S.B. (December 2013). Classification of robotic data using artificial neural network. Intelligent Computational Systems (RAICS), 2013 IEEE Recent Advances in (pp. 333–337).

[67] Kaminski W., Skrzypski J., Jach-Szakiel E. (2008, August). Application of artificial neural networks (ANNs) to predict air quality classes in big cities. 19th International Conference on Systems Engineering (pp. 135–140).

[68] Wang S.C., Dong J.X., Shen G. (1993). ANN-based process control in manufacturing. American Control Conference.

[69] Le T., Pardo P., Claster W. (2016). Application of artificial neural network in social media data analysis: a case of lodging business in Philadelphia. Artificial Neural Network Modelling (pp. 369–376).

[70] Barni M., Pelagotti A., Piva A. (2005). Image processing for the analysis and conservation of paintings: opportunities and challenges. IEEE Signal Process. Mag..

[71] He S., Li X. (September 2008). Application of a group search optimization based artificial neural network to machine condition monitoring. Emerging Technologies and Factory Automation, 2008. ETFA 2008. IEEE International Conference on (pp. 1260–1266).

[72] Barto A.G., Sutton R.S., Anderson C.W. (1983). Neuronlike adaptive elements that can solve difficult learning control problems. IEEE Trans. Syst. Man Cybern..

[73] Bogdan M., Schroder M., Rosenstiel W. (March 2003). Artificial neural net based signal processing for interaction with peripheral nervous system. Neural Engineering, 2003. Conference Proceedings. First International IEEE EMBS Conference on (pp. 134–137).

[74] Delinchant B., Duret D., Estrabaut L., Gerbaud L., Nguyen Huu H., Du Peloux B., Wurtz F. (2007). An optimizer using the software component paradigm for the optimization of engineering systems. COMPEL Int. J. Comput. Math. Electr. Electron. Eng..

[75] Janocha K., Czarnecki W.M. (2017). On Loss Functions for Deep Neural Networks in Classification.

[76] Yu S., Chi G. (2017). Weight optimization model based on the maximum discriminating power of credit evaluation result. Proceedings of the International Conference on Business and Information Management (pp. 6–11).

[77] Gogate M.R. (2017). New paradigms and future critical directions in heterogeneous catalysis and multifunctional reactors. Chem. Eng. Commun..

[78] Bram V. (1999). Future Perspectives in Catalysis, NRSC-catalysis Dutch National Research School Combination Catalysis Controlled by Chemical Design.

[79] Li H., Zhang Z., Liu Z. (2017). Application of artificial neural networks for catalysis: a review. Catalysts.

[80] Corma A., Serra J.M., Argente E., Botti V., Valero S. (2002). Application of artificial neural networks to combinatorial catalysis: modeling and predicting ODHE catalysts. ChemPhysChem.

[81] Behler J., Parrinello M. (2007). Generalized neural-network representation of high-dimensional potential-energy surfaces. Phys. Rev. Lett..

[82] Li H., Zhang Z. (2018). Mining the intrinsic trends of CO2 solubility in blended solutions. J. CO_2_ Util..

[83] Kalogirou S.A. (2001). Artificial neural networks in renewable energy systems applications: a review. Renew. Sustain. Energy Rev..

[84] Li H., Liu Z. (2018). Performance prediction and optimization of solar water heater via a knowledge-based machine learning method. Handbook of Research on Power and Energy System Optimization (pp. 55–74).

[85] Duch W., Diercksen G.H. (1994). Neural networks as tools to solve problems in physics and chemistry. Comput. Phys. Commun..

[86] Lynch M., Patel H., Abrahamse A., Rajendran A.R., Medsker L. (2001). Neural network applications in physics. Proceedings. IJCNN'01. International Joint Conference on Neural Networks (Vol. 3, pp. 2054–2058).

[87] Negoita G.A., Luecke G.R., Vary J.P., Maris P., Shirokov A.M., Shin I.J., Yang C. (2018). Deep Learning: a Tool for Computational Nuclear Physics.

[88] Choon L.S., Samsudin A., Budiarto R. (April 2004). Lightweight and cost-effective MPEG video encryption. Information and Communication Technologies: from Theory to Applications, 2004. Proceedings. 2004 International Conference on (pp. 525–526).

[89] Cho D., Tai Y.W., Kweon I.S. (2018). Deep convolutional neural network for natural image matting using initial alpha mattes. IEEE Transactions on Image Processing.

[90] Mayer J., Borges P.V., Simske S.J. (2018). Introduction. Fundamentals and Applications of Hardcopy Communication (pp. 1–5).

[91] Almazrooie M., Samsudin A., Gutub A.A.A., Salleh M.S., Omar M.A., Hassan S.A. (2018). Computer and Information Sciences.

[92] Narendra K.S., Parthasarathy K. (1990). Identification and control of dynamical systems using neural networks. IEEE Trans. Neural Network..

[93] LeCun Y., Bengio Y., Hinton G. (2015). Deep learning. Nature.

[94] Shi B., Bai X., Liu W., Wang J. (2014). Deep Regression for Face Alignment.

[95] Yang W.J., Chen Y.C., Chung P.C., Yang J.F. (2018). Multi-feature shape regression for face alignment. EURASIP J. Adv. Signal Proc..

[96] Wu Y., Ji Q. (2015). Discriminative deep face shape model for facial point detection. Int. J. Comput. Vis..

[97] Dominic S., Aparna C., Nath A.S., Mohan M., Ajeesh M.S., Antony A. (March 2016). A review of face detection system. Electrical, Electronics, and Optimization Techniques (ICEEOT), International Conference on (pp. 3536–3539).

[98] Toufiq R., Islam M.R. (2014). Face recognition system using PCA-ANN technique with feature fusion method. International Conference on Electrical Engineering and Information & Communication Technology (ICEEICT).

[99] Joseph S., Sowmiya R., Thomas R.A., Sofia X. (2014). Face detection through neural network. 2nd International Conference on Current Trends in Engineering and Technology (ICCTET).

[100] Oludare A.I., Jantan A., Omolara A.E., Singh M.M., Mohammed A., Kemi D.V. (July 2018). Terrorism prevention: a mathematical model for assessing individuals with profiling. Int. J. Comput. Sci. Network Secur..

[101] Cao C., Liu F., Tan H., Song D., Shu W., Li W., Xie Z. (2018). Deep learning and its applications in biomedicine. Genom. Proteom. Bioinform..

[102] Qi H., Chen C., Zhang H., Long J.J., Chung W.K., Guan Y., Shen Y. (2018). MVP: Predicting Pathogenicity of Missense Variants by Deep Learning.

[103] Lo J.Y., Floyd C.E. (1999). Application of artificial neural networks for diagnosis of breast cancer. CEC 99. Proceedings of the 1999 Congress on Evolutionary Computation (Vol. 3, pp. 1755–1759).

[104] Janghel R.R., Shukla A., Tiwari R., Kala R. (June 2010). Breast cancer diagnosis using artificial neural network models. 3rd International Conference on Information Sciences and Interaction Sciences (ICIS).

[105] Mehdy M.M., Ng P.Y., Shair E.F., Saleh N.I., Gomes C. (2017). Artificial neural networks in image processing for early detection of breast cancer. Comput. Math. Method. Med..

[106] Yue W., Wang Z., Chen H., Payne A., Liu X. (2018). Machine learning with applications in breast cancer diagnosis and prognosis. Designs.

[107] Oludare A.I., Jantan A., Omolara A.E., Singh M.M., Anbar M., Zaaba Z.F. (July, 2018). Forensic DNA profiling for identifying an individual crime. Int. J. Civil Eng. Technol. (IJCIET).

[108] Omolara A.E., Jantan A., Abiodun O.I., Singh M.M., Anbar M., Kemi D.V. (2018). State-of-the-art in big data application techniques to financial crime: a survey. Int. J. Comput. Sci. Network Secur..

[109] Xin Y., Kong L., Liu Z., Chen Y., Li Y., Zhu H., Wang C. (2018). Machine learning and deep learning methods for cybersecurity. IEEE Access.

[110] Dai Y., Wang G. (2018). Analyzing tongue images using a conceptual alignment deep autoencoder. IEEE Access.

[111] Hatcher W.G., Yu W. (2018). A survey of deep learning: platforms, applications and emerging research trends. IEEE Access.

[112] Mohammadi M., Al-Fuqaha A., Sorour S., Guizani M. (2018). Deep learning for IoT big data and streaming analytics: a survey. IEEE Commun. Surv. Tutorials.

[113] Qureshi I. (2015). Glaucoma detection in retinal images using image processing techniques: a survey. Int. J. Adv. Netw. Appl..

[114] Choi J.Y., Yoo T.K., Seo J.G., Kwak J., Um T.T., Rim T.H. (2017). Multi-categorical deep learning neural network to classify retinal images: a pilot study employing small database. PLoS One.

[115] Santos-García G., Galilea E.H. (2011). Using artificial neural networks to identify glaucoma stages. The Mystery of Glaucoma.

[116] Pierson H.A., Gashler M.S. (2017). Deep learning in robotics: a review of recent research. Adv. Robot..

[117] Olatunji I.E. (2018). Human Activity Recognition for Mobile Robot.

[118] Ruiz-del-Solar J., Loncomilla P., Soto N. (2018). A Survey on Deep Learning Methods for Robot Vision.

[119] He W., Yan Z., Sun Y., Ou Y., Sun C. (2018). Neural-learning-based control for a constrained robotic manipulator with flexible joints. IEEE Trans. Neural Network. Learn. Syst..

[120] Yang X., He H. (2018). Self-learning robust optimal control for continuous-time nonlinear systems with mismatched disturbances. Neural Network..

[121] Chen B.H., Huang S.C., Li C.Y., Kuo S.Y. (2017). Haze removal using radial basis function networks for visibility restoration applications. IEEE Trans. Neural Network. Learn. Syst..

[122] Li J., Mei X., Prokhorov D., Tao D. (2017). Deep neural network for structural prediction and lane detection in traffic scene. IEEE Trans. Neural Network. Learn. Syst..

[123] Mamuda M., Sathasivam S. (2017). Predicting the survival of diabetes using neural network. AIP Conf. Proc..

[124] Liebergen B.V. (Apr 15, 2017). Machine learning: a revolution in risk management and compliance. Inst. Int. Financ. J. Financ. Transform..

[125] Pandey A., Mishra A. (2017). Application of artificial neural networks in yield prediction of potato crop. Russ. Agric. Sci..

[126] Zhang X., Zhuang Y., Hu H., Wang W. (2017). 3-D Laser-based multiclass and multiview object detection in cluttered indoor scenes. IEEE Trans. Neural Network. Learn. Syst..

[127] Turner A.P., Caves L.S., Stepney S., Tyrrell A.M., Lones M.A. (2017). Artificial epigenetic networks: automatic decomposition of dynamical control tasks using topological self-modification. IEEE Trans. Neural Network. Learn. Syst..

[128] Nasir J., Yoo Y.H., Kim D.H., Kim J.H. (2017). User preference-based dual-memory neural model with memory consolidation approach. IEEE Trans. Neural Network. Learn. Syst..

[129] Deng Y., Bao F., Kong Y., Ren Z., Dai Q. (2017). Deep direct reinforcement learning for financial signal representation and trading. IEEE Trans. Neural Network. Learn. Syst..

[130] Radziszewski K. (2017). Artificial neural networks as an architectural design tool-generating new detail forms based on the Roman Corinthian order capital. IOP Conference Series: Materials Science and Engineering (Vol. 245, No. 6, p. 062030).

[131] Ansari A., Riasi A. (2016). Modelling and evaluating customer loyalty using neural networks: Evidence from startup insurance companies. Future Bus. J..

[132] Lotko A., Korneta P.A., Lotko M.A., Longwic R. (2018). Using Neural Networks in Modeling Customer Loyalty in Passenger Cars Maintenance and Repair Services. Appl. Sci..

[133] Qiu M., Song Y., Akagi F. (2016). Application of artificial neural network for the prediction of stock market returns. The case of the Japanese stock market. Chaos Solit. Fractals.

[134] Göçken M., Özçalıcı M., Boru A., Dosdoğru A.T. (2016). Integrating metaheuristics and artificial neural networks for improved stock price prediction. Expert Syst. Appl..

[135] Lee K.Y., Chung N., Hwang S. (2016). Application of an artificial neural network (ANN) model for predicting mosquito abundances in urban areas. Ecol. Inf..

[136] Pater Ł. (2016). Application of Artificial Neural Networks and Genetic Algorithms for Crude Fractional Distillation Process Modeling.

[137] Lai G., Liu Z., Zhang Y., Chen C.P. (2016). Adaptive position/attitude tracking control of aerial robot with unknown inertial matrix based on a new robust neural identifier. IEEE Trans. Neural Network. Learn. Syst..

[138] Onotu P., Day D., Rodrigues M.A. (3 – 6, May 2015). Accurate shellcode recognition from network traffic data using artificial neural nets. Electrical and Computer Engineering (CCECE), Sheffield Hallam University Sheffield, UK, 2015 IEEE.

[139] Gilmore R., Hanley N., O'Neill M. (May 2015). Neural network-based attack on a masked implementation of AES. Hardware-oriented Security and Trust, 2015 IEEE.

[140] Taro I., Donald E., Liu Y., Tetsuya O., Leonard B., Kazunori U. (24–27 March 2015). Application of neural networks for intrusion detection in tor networks. Advanced Information Networking and Applications Workshops (WAINA) 2015 IEEE.

[141] Gaikwad D.P., Thool R.C. (February. 2015). Intrusion detection system using bagging ensemble method of machine learning. Computing Communication Control and Automation (ICCUBEA), 2015 International Conference on.

[142] Kotenko I., Saenko I., Skorik F., Bushuev S. (May 2015). Neural network approach to forecast the state of the internet of things elements. Soft Computing and Measurements, XVIII International Conference.

[143] Hajdarevic A., Dzananovic I., Banjanovic-Mehmedovic L., Mehmedovic F. (May 2015). Anomaly detection in thermal power plant using probabilistic neural network. Information and Communication Technology, Electronics and Microelectronics (MIPRO), 2015 38th International Convention.

[144] Zhukov A., Tomin N., Sidorov D., Panasetsky D., Spirayev V. (May 2015). A hybrid artificial neural network for voltage security evaluation in a power system. Energy (IYCE), 2015 5th International Youth Conference.

[145] Santosh T.V., Vinod G., Saraf R.K., Ghosh A.K., Kushwaha H.S. (2007). Application of artificial neural networks to nuclear power plant transient diagnosis. Reliab. Eng. Syst. Saf..

[146] Huang S., Yu F., Tsaih R., Huang Y. (July 2015). Network-traffic anomaly detection with incremental majority learning. Neural Networks 2015 International Joint Conference.

[147] Turčanik M. (May 2015). Packet filtering by artificial neural network. Military Technologies, 2015 International Conference.

[148] Fatima H., Al-Turki S.M., Pradhan S.K., Dash G.,N. (March 2015). Information security: artificial immune detectors in neural networks. Web Applications and Networking (WSWAN), 2015 2nd World Symposium on.

[149] Kitchens F., Harris T. (December, 2015). Genetic adaptive neural networks for prediction of insurance claims. Int. J. Eng. Adv. Res. Technol. (IJEART).

[150] Zeinalizadeh N., Shojaie A.A., Shariatmadari M. (2015). Modeling and analysis of bank customer satisfaction using neural networks approach. Int. J. Bank Market..

[151] Abbinaya S., Kumar M.S. (April 2015). Software effort and risk assessment using decision table trained by neural networks. Communications and Signal Processing (ICCSP), 2015 International Conference, pp. 1389–1394.

[152] Gupta S., Kashyap S. (2015). Forecasting inflation in G-7 countries: an application of artificial neural network. Foresight.

[153] Zhong X., He H., Zhang H., Wang Z. (2015). A neural network based online learning and control approach for Markov jump systems. Neurocomputing.

[154] Dahikar S.S., Rode S.V. (2014). Agricultural crop yield prediction using artificial neural network approach. Int. J. Innovat. Res. Electr. Electron. Instrum. Contr. Eng..

[155] Zhu J., Liu S. (March 21, 2014). SOM network-based clustering analysis of real estate enterprises. Am. J. Ind. Bus. Manag..

[156] Igor H., Bohuslava J., Martin J., Martin N. (2014). Application of neural networks in computer security. Proc. Eng..

[157] Ecer F. (2013). Comparing the bank failure prediction performance of neural networks and support vector machines: the Turkish case. Ekonomska Istraživanja.

[158] Bahia I.S.H. (2013). Using artificial neural network modeling in forecasting revenue: case study in national insurance company/Iraq. Int. J. Intell. Sci..

[159] Khaze S.R., Masdari M., Hojjatkhah S. (2013). Application of Artificial Neural Networks in Estimating Participation in Elections.

[160] Kareem E.I.A., Alsalihy W.A.A., Jantan A. (2012). Multi-connect architecture (MCA) associative memory: a modified hopfield neural network. Intell. Autom. Soft Comput..

[161] Nasr M.S., Moustafa M.A.E., Seif H.A.E., El Kobrosy G. (2012). Application of artificial neural network (ANN) for the prediction of EL-AGAMY wastewater treatment plant performance-Egypt. Alexandria Eng. J..

[162] Adebiyi A., Arreymbi J., Imafidon C. (2012). Assessment of software design using neural network. School of Architecture, Computing and Engineering University of East London, London, UK. Int. J. Adv. Res. Artif. Intell. (IJARAI).

[163] Cheng F., Sutariya V., Cheng F., Sutariya V. (2012). Artificial neural networks: a new paradigm for thermal science and engineering. College of Pharmacy, University of South Florida, USA. Clin. Exp. Pharmacol..

[164] Korany M.A., Mahgoub H., Fahmy O.T., Maher H.M. (2012). Application of artificial neural networks for response surface modelling in HPLC method development. J. Adv. Res..

[165] Cheng F., Sutariya V. (2012). Applications of artificial neural network modeling in drug discovery. College of Pharmacy, University of South Florida, USA. Clin. Exp. Pharmacol..

[166] Stahl B., Carroll-Mayer M., Elizondo D., Wakunuma K., Zheng Y. (2012). Intelligence Techniques in Computer Security and Forensics: at the Boundaries of Ethics and Law.

[167] Yan W. (2012). Toward automatic time-series forecasting using neural networks. IEEE Trans. Neural Network. Learn. Syst..

[168] Cao Y., He H., Huang H.H. (2011). LIFT: a new framework of learning from testing data for face recognition. Neurocomputing.

[169] Junior J.L., Neto A.J.D., Neto L.B., Soeiro F.J.C.P., Santana C.C., Velho H.F.C. (2011). Application of artificial neural networks and hybrid methods in the solution of inverse problems. Artificial Neural Networks - Application. In Tech. April.

[170] Quetglas A., Ordines F., Guijarro B. (2011). The use of artificial neural networks (ANNs) in aquatic ecology. Artificial Neural Networks - Application.

[171] Šťastný J., Konečný V., Trenz O. (2011). Agricultural data prediction by means of neural network. Agric. Econ..

[172] Mishra A.K., Ramesh L. (April 2009). Application of neural networks in wind power (generation) prediction. Sustainable Power Generation and Supply.

[173] Webb R., Doble P., Dawson M. (2009). Optimisation of HPLC gradient separations using artificial neural networks (ANNs): application to benzodiazepines in post-mortem samples. J. Chromatog..

[174] Shanthi D., Sahoo G., Saravanan N. (2009). Designing an artificial neural network model for the prediction of thrombo-embolic stroke. J. Biometric Bioinf. (IJBB).

[175] Patel J.L., Goyal R.K. (September 2007). Applications of artificial neural networks in medical science. Curr. Clin. Pharmacol..

[176] Liu Z., Guan X., Wu H. (2006). Bandwidth prediction and congestion control for ABR traffic based on neural networks. Adv. Neural Network..

[177] Wen J., Dai Q., Jin Y. (2006). A neural network decision-making mechanism for robust video transmission over 3G wireless network,” Department of Automation, Tsinghua University, Beijing 100084, China. Adv. Neural Network..

[178] Grediaga Á., Ibarra F., García F., Ledesma B., Brotóns F. (2006). Application of neural networks in network control and information security. Adv. Neural Network..

[179] Zhu S., Zhang Y., Lu X. (2006). Detection for triangle traffic sign based on neural network. International Symposium on Neural Networks.

[180] Dong-Chul P.N., Yunsik L. (May, 2006). Multiscale bilinear recurrent neural networks and their application to the long-term prediction of network traffic. International Symposium on Neural Networks.

[181] Rigol-Sanchez J.P., Chica-Olmo M., Abarca-Hernandez F. (2003). Artificial neural networks as a tool for mineral potential mapping with GIS. Int. J. Rem. Sens..

[182] Agatonovic-Kustrin S., Beresford R. (2000). Basic concepts of artificial neural network (ANN) modeling and its application in pharmaceutical research. J. Pharmaceut. Biomed..

[183] Kate A.S., Gupta J.N.D. (2000). Neural networks in business: techniques and applications for the operations researcher. Comput. Oper. Res..

[184] Pasika H., Haykin S., Clothiaux E., Stewart R. (1999). Neural networks for sensor fusion in remote sensing. Neural Networks, International Joint Conference on 4.

[185] Fanning K.M., Cogger K.O. (1998). Neural network detection of management fraud using published financial data. Int. J. Intell. Syst. Account. Finance Manag..

[186] Cannady J. (October 1998). Artificial neural networks for misuse detection.

[187] Tan C.N. (1997). An Artificial Neural Networks Primer with Financial Applications Examples in Financial Distress Predictions and Foreign Exchange Hybrid Trading System.

[188] Senator T.E., Goldberg H.G., Wooton J., Cottini M.A., Khan A.U., Klinger C.D., Wong R.W. (1995). Financial crimes enforcement network AI system (FAIS) identifying potential money laundering from reports of large cash transactions. AI Mag..

[189] Dony R.D., Haykin S. (1995). Neural network approaches to image compression. Proc. IEEE.

[190] Lin C.T., Lee C.S.G. (1991). Neural-network-based fuzzy logic control and decision system. IEEE Trans. Comput..

[191] Jiang H., Zhang H. (2018). Iterative ADP learning algorithms for discrete-time multi-player games. Artif. Intell. Rev..

[192] Wang D., Liu D., Zhao D., Huang Y., Zhang D. (2013). A neural-network-based iterative GDHP approach for solving a class of nonlinear optimal control problems with control constraints. Neural Comput. Appl..

[193] Shi G., Wei Q., Liu D. (2017). Optimization of electricity consumption in office buildings based on adaptive dynamic programming. Soft Comput..

[194] Besseris G.J. (2018). Taguchi-generalized regression neural network micro-screening for physical and sensory characteristics of bread. Heliyon.

[195] Nishio M., Nagashima C., Hirabayashi S., Ohnishi A., Sasaki K., Sagawa T., Yamashita T. (2017). Convolutional auto-encoder for image denoising of ultra-low-dose CT. Heliyon.

[196] Oludare A.I., Jantan A., Abiodun E.O., Singh M.M., Abubakar Z.L., Umar A.M. (14–16, March 2018). Big data: an approach for detecting terrorist activities with people’s profiling. Proceedings of the International MultiConference of Engineers and Computer Scientists, Hong Kong, Vol I IMECS 2018.

[197] Mahmadi F.N., Zaaba Z.F., Samsudin A. (2017). Security issues in online banking: review cases in Malaysia. Adv. Sci. Lett..

[198] Abiodun E.O., Jantan A., Oludare A.I., Poston H.E. (14–16, March 2018). A novel approach for the adaptation of honey encryption to support natural language message. Proceedings of the International Multi-conference of Engineers and Computer Scientists, Vol I IMECS 2018.

[199] Omolara A.E., Jantan A., Oludare A.I., Arshad H. (2018). An enhanced practical difficulty of one-time pad algorithm resolving the key management and distribution problem. Proceedings of the International Multi-conference of Engineers and Computer Scientists.

[200] Felix G., Nápoles G., Falcon R., Froelich W., Vanhoof K., Bello R. (2017). A review on methods and software for fuzzy cognitive maps. Artif. Intell. Rev..

[201] Dhanalakshmi S., Subramanian C. (2014). An analysis of data mining applications for fraud detection in securities market. J. Data Mining Techn. Appl..

[202] J. Ahire, Artificial Neural Networks: the Brain behind AI. Lulu. com.

[203] Medler D.A. (1998). A brief history of connectionism. Neural Comput. Surv..

[204] Gallant S.I. (1998). Connectionist expert systems. Commun. ACM.

