# Supplementary material for: State-of-the-art in artificial neural network applications: A survey
Source: Heliyon. 2018 Nov 23;4(11):e00938. doi: 10.1016/j.heliyon.2018.e00938 (PMC6260436; doi:10.1016/j.heliyon.2018.e00938)
Supplement: Supplementary Table 1 [file mmc1.docx]

Supplementary Table 1

| [**Financial**](http://www.alyuda.com/products/forecaster/applications1.htm) | **Science** | **Science** | [**Medical**](http://www.alyuda.com/products/forecaster/applications3.htm) | **Energy** | **Industry/ Manufacturing** |
| --- | --- | --- | --- | --- | --- |
| [Property appraisal](http://www.alyuda.com/products/forecaster/applications1.htm#a5)  prediction | Physical system modeling | Recognizing genes | [Detection of medical phenomena](http://www.alyuda.com/products/forecaster/applications3.htm#a2) | Energy demand forecasting | [Temperature and force prediction](http://www.alyuda.com/products/forecaster/applications5.htm#a3) |
| Economic indicator forecast | Chemical compound identification | Polymer identification | [Medical diagnosis](http://www.alyuda.com/products/forecaster/applications3.htm#a1) | Predicting gas/coal index prices | [Quality control](http://www.alyuda.com/products/forecaster/applications5.htm#a2) |
| Stock market prediction | Optimization | Ecosystem Evaluation | Medical phenomena | Electrical Load Forecasting | [Managerial decision making](http://www.alyuda.com/products/forecaster/applications4.htm#a3) |
| [Creditworthiness](http://www.alyuda.com/products/forecaster/applications1.htm#a2) | Recipes and Chemical Formulation | Ground Level Ozone Prognosis | [Treatment cost estimation](http://www.alyuda.com/products/forecaster/applications3.htm#a4) | Short term and long term load estimation | [Retail inventories optimization](http://www.alyuda.com/products/forecaster/applications4.htm#a1) |
| [Price forecasts](http://www.alyuda.com/products/forecaster/applications1.htm#a7) | Pattern recognition | Odor analysis and identification | Patient’s length of staying forecasts | Hydro dam monitoring | Process modeling and analysis |
| Fraud detection | Signal processing, neural filtering | Biological systems analysis | Pattern recognition | Power control systems | Production cost prediction |
| [Bankruptcy prediction](http://www.alyuda.com/products/forecaster/applications1.htm#a4) | Botanical classification | Signal processing | Prediction | Power output and input prediction | Machine pressure prediction |
| Portfolio trading, | Prediction | Prediction | Prediction | Nuclear energy life span prediction | Equipment reliability  prediction |
| [Credit rating](http://www.alyuda.com/products/forecaster/applications1.htm#a3) | Data validation | Neural filtering | [Evaluation of medical condition](http://www.alyuda.com/products/forecaster/applications3.htm#a2) | Mechanical material modelling | Data validation |
| Investment support | Computer, and computational intelligence | Space technology and data validation | Disease and epidemics prediction | Data validation |  |
| Credit and policy approval | Bomb | Prediction | Prediction | Modeling and prediction | Quality and composition prediction |
| Customer research | Iceberg | Dynamic load shedding | Classification | Soil fertility prediction | Modelling for diverse uses of water |
| Fraud detection | Fraud detection | Pattern recognition | Production estimates | Animals and crop yield prediction | Pattern recognition |
| Prediction | Data validation | Data validation | Pattern recognition | Pattern recognition | Classification |

Table 1: ANNs model applications to different areas regarding prediction, pattern recognition, and classification.

|  |  |  |  |  |  |
| --- | --- | --- | --- | --- | --- |
| **Educational** | [**Sales and Marketing**](http://www.alyuda.com/products/forecaster/applications2.htm) | [**Data Mining**](http://www.alyuda.com/products/forecaster/applications7.htm) | [**HR Management**](http://www.alyuda.com/products/forecaster/applications6.htm) | [**Operational Analysis**](http://www.alyuda.com/products/forecaster/applications4.htm) | **Games** |
| Predict student performance | [Service usage forecasting](http://www.alyuda.com/products/forecaster/applications2.htm#a3) | Time series analysis | [Staff scheduling](http://www.alyuda.com/products/forecaster/applications6.htm#a3) | [Cash flow forecasting](http://www.alyuda.com/products/forecaster/applications4.htm#a4) | Games development |
| College application screening | [Sales forecasting](http://www.alyuda.com/products/forecaster/applications2.htm#a1) | [Classification](http://www.alyuda.com/products/forecaster/applications7.htm#a2) | [Employee retention](http://www.alyuda.com/products/forecaster/applications6.htm#a2) | [Scheduling optimization](http://www.alyuda.com/products/forecaster/applications4.htm#a2) | Sports betting and making horse |
| Teaching neural networks | [Retail margins forecasting](http://www.alyuda.com/products/forecaster/applications2.htm#a4) | [Change and deviation detection](http://www.alyuda.com/products/forecaster/applications7.htm#a2) | [Employee selection and hiring](http://www.alyuda.com/products/forecaster/applications6.htm#a1) | [Service usage forecasting](http://www.alyuda.com/products/forecaster/applications2.htm#a3) | Sports betting and making horse |
| Neural network research | [Targeted marketing](http://www.alyuda.com/products/forecaster/applications2.htm#a2) | [Response modeling](http://www.alyuda.com/products/forecaster/applications7.htm#a2) | [Retail margins forecasting](http://www.alyuda.com/products/forecaster/applications2.htm#a4) | Pattern recognition | Dog racing picks |
| Pattern recognition | Pattern recognition | [Knowledge discovery](http://www.alyuda.com/products/forecaster/applications7.htm#a2) | Pattern recognition | [Classification](http://www.alyuda.com/products/forecaster/applications7.htm#a2) | Modelling |
| Modelling | Modelling | [Prediction](http://www.alyuda.com/products/forecaster/applications7.htm#a1), Data validation | Modelling | Prediction | Pattern recognition |
| Prediction | Prediction | Prediction | Prediction | classification | Wining prediction |
|  |  |  |  |  |  |
| Policy | **Management** | **Military** | **Production** | **Climate and Weather** | **Other** |
| Political election prediction | Risk management | Land, water and air operation | Agricultural production estimates | Quantitative weather forecasting | Optimization methods |
| Pattern recognition | Prediction | Enemies location prediction | Industrial production estimates | Sun shine temperature prediction | Detection applications |
| Fraud detection | Control | Success target prediction | Manpower prediction | Wind and rainfall, pattern recognition | Transportation modelling and travel prediction |
| Classification | Fuzzy logic control and decision systems | Weapon effectiveness | Life span of equipment modelling | Cloud formation prediction and weather,  climate change  prediction | Art works modelling and pattern recognition |
|  |  |  |  |  |  |

Table 1: Continue
