# Supplementary material for: State-of-the-art in artificial neural network applications: A survey
Source: Heliyon. 2018 Nov 23;4(11):e00938. doi: 10.1016/j.heliyon.2018.e00938 (PMC6260436; doi:10.1016/j.heliyon.2018.e00938)
Supplement: Supplementary Table 2 [file mmc2.docx]

Supplementary Table 2

Table 2: Comparison of different ANN models, application areas and contribution

| **No** | **Author(s)/year** | **Modelling** | **ANN Application Area** | **Contribution to knowledge** |
| --- | --- | --- | --- | --- |
| **1** | W. He, Z. Yan, Y. Sun, Y. Ou, C. Sun, (2018) [120] | Prediction | Robot control | The result shows the effectiveness of using ANN control on flexible joint manipulator model. |
| **2** | X. Yang, H. He, (2018) [121] | Prediction | Optimization | The optimal control scheme design demonstrates that feedback controller optimizes a specified value function. |
| **3** | B. H. Chen, S. C. Huang, C. Y. Li, S. Y. Kuo, (2017) [122] | Prediction | Weather | The ANN application effectively removes haze formation and restore visibility and brightness in hazy images. |
| **4** | J. Li , X. Mei, D. Prokhorov, D. Tao, (2017) [123] | Pattern recognition | Traffic scene lane detection | The result shows that recurrent neural network automatically detects lane boundaries. |
| **5** | M. Mamuda, S. Sathasivam, (2017) [124] | Prediction | Medical science | The result demonstrates that Levenberg-Marquardt algorithm produces mean square of error as 0.00025091 and R-square of 1. Meaning that there is a good correlation between the target input and predicted output. |
| **6** | B. V. Liebergen, (2017) [125] | Pattern recognition | Finance auditing | The result demonstrates that machine learning models improve the auditing. |
| **7** | A. Pandey, A. Mishra, (2017) [126] | Prediction | Agricultural yield of potato crops | Based on lower spread constant (0.5) and quick learning capability the GRNN perform a better predictor more than RBFNN. Also, the rough surface field was proving to be more productive than flat field. |
| **8** | X. Zhang, Y. Zhuang, H. Hu, W. Wang, (2017) [127] | Pattern recognition/  Prediction | Security | Experimental results show that proposed method can be apply to many 2-D object detection tasks with fewer training data. |
| **9** | P. Turner, L. S. Caves, S. Stepney, A. M. Tyrrell, M. A. Lones, (2017) [128] | Classification | Computational models | The result shows the potentiality of self-modifying processes within ANN connectionist architectures. |
| **10** | J. Nasir, Y. H. Yoo, D. H. Kim, J. H. Kim, (2017) [129] | Pattern recognition/  Prediction | Memory modeling | The results verify different accurate learning of the neural model with higher user preference based dual memory neural model. |
| **11** | Y. Deng, F. Bao, Y. Kong, Z. Ren, Q. Dai, (2017) [130] | Prediction | Stock and commodity market | The result demonstrates that neural system can be robust in predicting the future of stock and the commodity markets under broad testing conditions |
| **12** | K. Radziszewski, (2017) [131] | Prediction | Architectural design  prediction | The result demonstrates fast computing tool for architectural design. |
| **13** | 1. [Azarnoush,](http://www.sciencedirect.com/science/article/pii/S2314721016300019#!) R. [Arash,](http://www.sciencedirect.com/science/article/pii/S2314721016300019#!) (2016) [132] | Pattern recognition/  Prediction | Customer loyalty | The result demonstrates that neural networks can analyzed perceived value, customer loyalty, and customer satisfaction than any methods. |
| **14** | M. Qiu, Y. Song, F. Akagi, (2016) [133] | Prediction | Stock market prediction | Experiments result show effectiveness of the selected input parameters to predicting stock market profits. |
| **15** | M. Göçken, M. Özçalıcı, A. Boru A. T. Dosdoğru, (2016) [134] | Prediction | Stock market prices prediction | The result demonstrates forecasting performance of the ANN i.e. mean absolute percentage error (MAPE) = 3.38 is significantly better than genetic algorithm (GA) ANN which has MAPE of 3.86 and MAPE of 3.81 respectively. |
| **16** | K. Y. Lee, N. Chung, S. Hwang, (2016) [135] | Pattern recognition/  Prediction | Medical science research | The result indicates that the ANN model was able to predict the high variability as compared to MLR method. |
| **17** | Ł. Pater, (2016) [136] | Classification/ Prediction | Crude oil production and fractions quality prediction | The results show that neural networks used in together with genetic algorithms prove to be accurate prediction fractions quality. |
| **18** | G. Lai, Z. Liu, Y. Zhang, C. P. Chen, (2016) [137] | Pattern recognition/ Prediction | Military in air operation | The proposed controller provides simulation, implementation and prediction of performance in military air operation. |
| **19** | P. Onotu, D. Day, M. A. Rodrigues, (2015) [138] | Pattern recognition | Software security | The proposed ANN method result shows an accuracy of 100%. |
| **20** | R. Gilmore, N. Hanley, M. O’Neill, (2015) [139] | **Classification** | Computer and information and communication security | The result show that neural networks provide for efficient classification and a robust in the context of side-channel attacks. |
| **21** | I. Taro, E. Donald, Y. Liu, O. Tetsuya, B. Leonard, U. Kazunori, (2015) [140] | Pattern recognition | Computer network security | The results show that the simulation system have good approximation and can be use for intrusion detection in Tor networks. |
| **22** | D. P. Gaikwad, R. C. Thool, (2015) [141] | Pattern recognition | Computer network security | The result demonstrates successes and low false positives compared to machine learning techniques. |
| **23** | I. Kotenko, I. Saenko, F. Skorik, S. Bushuev, **(2015) [142]** | **Prediction** | Internet Domain | The result indicates high efficiency of decision-making and accuracy of forecasting the states of elements of the internet of things. |
| **24** | A. Hajdarevic, I. Dzananovic, L. Banjanovic-Mehmedovic, F. Mehmedovic, (2015) [143] | Pattern recognition | Energy and power industrial applications | The results confirmed that probabilistic NN achieved signficant solution for anomaly detection problem in a real-time industrial application. |
| **25** | A. Zhukov, N. Tomin, D. Sidorov, D. Panasetsky, V. Spirayev, (2015)  [144] | Pattern recognition/  Prediction | Energy and power industrial applications | The results showed the capability of the hybrid ANN base models in performing an on-line security monitoring for power voltage. |
| **26** | T. V. Santosh, G. Vinod, R. K. Saraf, A. K. Ghosh, H. S. Kushwaha, (2015) [145] | Prediction | Nuclear energy | The result demonstrates that NPP performs better using resilient back propagation algorithm. |
| **27** | S. Huang, F. Yu, R. Tsaih, Y. Huang, (2015) [146] | Pattern recognition | Computer network security | The result reveals identification of samples that have abnormal features and positivity in detecting attacks on the network. |
| **28** | M. Turčanik, (2015) [147] | Pattern recognition/ Prediction | Computer network security | The result show high level optimization after simulation. |
| **29** | H. Fatima, S. M. Al-Turki, S. K. Pradhan, G, N, Dash, (2015) [148] | **Prediction** | Computer and information and communication security | The results indicated a high correlation coefficient and R-square values between the calculated and predicted output variables, up to 0.9 or 90%. |
| **30** | F. Kitchens, T. Harris, (2015) [149] | Prediction | Finance and insurance business | The result demonstrates a fraud detection process efficiently. |
| **31** | N. Zeinalizadeh, A. A. Shojaie, M. Shariatmadari, (2015) [150] | Pattern recognition/ Prediction | Bank and financial institutions customer’s satisfaction | The result show 73% higher accuracy for ANN model compared to the 27% accuracy of linear regression model at predicting overall bank customer satisfaction. |
| **32** | S, Abbinaya, M.S. Kumar, (2015) [151] | **Prediction** | Software security | The result shows that ANN provides accurate forecasts for the software developed. |
| **33** | S. Gupta, S. Kashyap, (2015) [152] | Prediction/forecasting | Government policy-makers on forecasting inflation | The results demonstrate forecasting inflation for economists, policy-makers, and practitioners. |
| **34** | X. Zhong, H. He, H. Zhang, Z. Wang, (2015) [153] | Classification | Education | The results demonstrate the effectiveness of the proposed technique. |
| **35** | S. S. Dahikar, S. V. Rode, (2014) [154] | Prediction | Agricultural crop yield prediction | The result shows that artificial neural networks powerful tools as it increases crop yield prediction more effectively and efficiently. |
| **36** | J. Zhu, S. Liu, (2014) [155] | Prediction | [Real estate enterprises](http://www.scirp.org/(S(i43dyn45teexjx455qlt3d2q))/journal/PaperInformation.aspx?PaperID=44048) | Experimental results show that NN comprehensively displayed two-dimensional graphics and prediction for financial system for each real estate enterprise. |
| **37** | H. Igor, J. Bohuslava, J. Martin, N. Martin, (2014) [156] | Pattern recognition/ Prediction | Computer security | The result demonstrates the effectiveness of control system data communication. |
| **38** | F. Ecer, (2013) [157] | Prediction | Bank failures prediction | The result demonstrates that neural network models are better predictors. |
| **39** | 1. S. H. Bahia, (2013) [158] | Prediction | Finance and insurance | The data analyses show approximately 120% growth indicator of insurance premiums revenue for 41 years. |
| **40** | S. R. Khaze, M. Masdari, S. Hojjatkhah, (2013) [159] | Prediction | Policy such as political election | The test result shows that anticipates participation rate of the public in Boyerahmad and kohgiloye province in the future presidential election of Iran with 91% accuracy. |
| **41** | E. I. A. Kareem, W. A. A. Alsalihy, A. Jantan, (2012) [160] | Pattern recognition/  Prediction | Soft computing | The result reveals improvement in Hopfield NN net architecture, learning and convergence |
| **42** | M. S. Nasr, M. A. E. Moustafa, H. A. E. Seif, G. El Kobrosy, (2012) [161] | Prediction | Engineering and Wastewater treatment | The result shows 0.90 or 90% prediction of the ANN plant performance with a correlation coefficient (R) between the predicted output and observed variables. |
| **43** | A. Adebiyi, J. Arreymbi, C. Imafidon, (2012) [162] | Prediction | Software security | **The result shows that** neural network can reduce cost and save time when security is to integrate during software development. |
| **44** | **F. Cheng, V. Sutariya,** (2012) [163] | Classification | Applications in engineering | The result indicates that ANN methods and the corresponding new paradigm represent a promising way to approach and solve difficult thermal problems. |
| **45** | M. A. Korany, H. Mahgoub, O. T. Fahmy, H. M. Maher, (2012) [164] | Prediction | Science research | The result show ANN predict more accurately than multiple regression model. |
| **46** | **F. Cheng, V. Sutariya,** (2012) [165] | Pattern recognition/  Prediction | Medical science | The result indicates that ANNs are powerful tools for drug discovery. |
| **47** | 1. Stahl, M. Carroll-Mayer, D. Elizondo, K. Wakunuma, Y. Zheng, (2012) [166] | Pattern recognition | Computer security and Forensics | The result highlights likely ANNs ethical and legal problems and area of future research. |
| **48** | W. Yan, (2012) [167] | Prediction | time-series forecasting | The result proves effective for modelling large-scale business time series. |
| **49** | Y. Cao, H. He, H. H. Huang, (2011) [168] | Pattern recognition | Nation security | Experimental results prove effectiveness of the proposed ANN framework. |
| **50** | J. L. Junior, A. J. D. Neto, L. B. Neto, F. J. C. P. Soeiro, C. C. Santana, H. F. C. Velho, (2011) [169] | Classification | Manufacturing/ industry | The result demonstrates a significant solution for the gas-liquid adsorption isotherm inverse challenge. |
| **51** | A. Quetglas, F. Ordines, B. Guijarro, (2011) [170] | Pattern recognition/  Prediction | Ecological science | The experimental result shows flexible and readily combination of methods. The result helps ecologists to develop complex models regarding the analysis of ecological systems. |
| **52** | J. Šťastný, V. Konečný, O. Trenz, (2011) [171] | Prediction | Agricultural crop yield prediction | The experimental result shows that multilayer NN more accurate in a task case than findings from the published regressive model. |
| **53** | A. K. Mishra, L. Ramesh, (2009)  [172] | Prediction | Wind power | The result of the neural network model shown a good agreement and produced the wind forecast with the accuracy more than 90%. |
| **54** | R. Webb, P. Doble, M. Dawson, (2009) [173] | Prediction | Medical science | The result indicates 0.0057 - 0.023 g/mL records limits of medical problem detection and medical problem recoveries between 58% and 92% accuracy. |
| **55** | D. Shanthi, G. Sahoo, N. Saravanan, (2009) [174] | Prediction | Medical science | The result proves that ANN model assists the doctors to plan better medication for patient. Also, the outcome provide patient with quality diagnosis. General predictive accuracy was 89%. |
| **56** | M. A. Boyacioglu, Y. Kara, Ö. K.  (2009) [175] | Prediction | Bank and financial business | The experimental result reveals learning in combined vector quantization and multilayer perceptron NN architectures better prediction of bank financial failure than other methods. |
| **57** | J. L. Patel, R. K. Goyal, (2007) [176] | Clustering | Medical science | The result demonstrates functionality of biological neural cluster fundamentally. |
| **58** | Z. Liu, X. Guan, H. Wu, (2006) [177] | Prediction | Computer and information security | The results predict good control of ABR service in ATM networks. |
| **59** | J. Wen, Q. Dai, Y. Jin, (2006) [178] | Pattern recognition/  Prediction | Computer, information and communication security | The result proves control for video transmission over 3G network. Even when an error exists. |
| **60** | Á. Grediaga, F. Ibarra, F. García, B. Ledesma, F. Brotóns, (2006) [179] | Pattern recognition | Computer and information security | A series of experimental result shown that neural network detects some complicated forms of attacks in a system and the result was better when compared to others found in literature. |
| **61** | S. Zhu, Y. Zhang, X. Lu, (2006) [180] | Pattern recognition | Transportation | The result shows that the percentage of correctness is 100%. |
| **62** | P. N. Dong-Chul, L. Yunsik, (2006) [181] | Prediction | Computer and information security | The result demonstrates creating a new wavelet-based neural network architecture for long-term prediction of network traffic. |
| **63** | J. P. Rigol-Sanchez, M. Chica-Olmo, F. Abarca-Hernandez, (2003) [182] | Classification/ clustering | Mining and mineral resources | The result demonstrates that an artificial neural network is an effective tool for mineral exploration spatial data modeling. |
| **64** | S. Agatonovic-Kustrin, R. Beresford, (2000) [183] | Pattern recognition/  Prediction | Pharmaceutical research | The result indicates that supervised associating networks can be applying in pharmaceutical fields as an alternative to conventional response surface methodology. |
| **65** | 1. S. Kate, J. N. D. Gupta, (2000) [184] | Clustering | Business and operations research | The result shows success application of neural networks in business and operation research. |
| **66** | H. Pasika, S. Haykin, E. Clothiaux, R. Stewart, (1999) [185] | Pattern recognition | Sensor fusion in remote sensing | Neural network application demonstrates success in making laser radar (LIDAR) output of cloud base height information available at a network of ground- based meteorological stations without necessarily installing LIDAR sensors. |
| **67** | K. M. Fanning, K. O. Cogger, (1998) [186] | Pattern recognition/  Prediction | Financial detecting management fraud | The result proves fraudulent of financial statements. |
| **68** | J. Cannady, (1998) [187] | Pattern recognition/  Prediction | Computer and information and communication security | A systematic process of identifying instances of network attacks by comparing current function or activity and the expected actions of an intruder. |
| **69** | C. N. Tan, (1997) [188] | Pattern recognition/  Prediction | Trading and financial destress prediction | The results indicate that ANNWAR model application could determine more profitable and robust trading system because it performs better covering a wider range of filter values than previous models. |
| **70** | T. E. Senator, H. G. Goldberg, J. Wooton, M. A. Cottini, A. U. Khan, C. D. Klinger, R. W. Wong, (1995) [189] | Pattern recognition | Finance and money laundering | The result demonstrates that neural network identifies potential money laundering from cash transactions. |
| **71** | R. D. Dony, S. Haykin, (1995)[190] | Pattern recognition/  Prediction | Signal procession and Computational intelligence | The application of neural networks to the problem of image compression produces promising results. |
| **72** | C. T. Lin, C. S. G. Lee, (1991) [191] | Prediction | Management | The result indicates that learning speed converges much faster than the original backpropagation learning algorithm with practical application. |
| **73** | H. Jiang, H. Zhang, (2018) [192] | Prediction | Optimization and programming | Simulation results demonstrate the effectiveness of the developed schemes. |
| **74** | D. Wang, D. Liu, D. Zhao, Y. Huang, D. Zhang, (2013) [193] | Prediction | Optimal control problem | Simulation verify the effectiveness of the control scheme in solving the constrained optimal control problem. |
| **75** | G. Shi, Q. Wei, D. Liu, (2017) [194] | Classification/  prediction | Electricity consumption | The model developed improve the electricity consumption as demonstrated. |
| **76** | G. J. Besseris, (2018) [195] | Prediction | Industrial operations | The result show that the proposed generalized regression neural networks (GRNN) intelligence and the concept of the Wisdom of Crowds play a significantly role in the oven-zone temperatures setting for concurrent screening-and the optimization was found to be 270–240 °C. |
| **77** | M. Nishio, C. Nagashima, S. Hirabayashi, A. Ohnishi, K. Sasaki, T. Sagawa, T. Yamashita, (2017) [196] | Prediction | Communication | The results of our proposed method were statistically better than those of block-matching and 3D filtering (p-values < 0.05). |
